# Prevention and control of dengue and chikungunya in Colombia: A cost-effectiveness analysis

**DOI:** 10.1371/journal.pntd.0010086

**Published:** 2021-12-29

**Authors:** Anneke L. Claypool, Margaret L. Brandeau, Jeremy D. Goldhaber-Fiebert

**Affiliations:** 1 Department of Management Science and Engineering, Stanford University, Stanford, California, United States of America; 2 Department of Health Policy, Stanford University, Stanford, California, United States of America; Oregon Health and Science University, UNITED STATES

## Abstract

**Background:**

Chikungunya and dengue are emerging diseases that have caused large outbreaks in various regions of the world. Both are both spread by *Aedes aegypti* and *Aedes albopictus* mosquitos. We developed a dynamic transmission model of chikungunya and dengue, calibrated to data from Colombia (June 2014 –December 2017).

**Methodology/Principal findings:**

We evaluated the health benefits and cost-effectiveness of residual insecticide treatment, long-lasting insecticide-treated nets, routine dengue vaccination for children aged 9, catchup vaccination for individuals aged 10–19 or 10–29, and portfolios of these interventions. Model calibration resulted in 300 realistic transmission parameters sets that produced close matches to disease-specific incidence and deaths. Insecticide was the preferred intervention and was cost-effective. Insecticide averted an estimated 95 chikungunya cases and 114 dengue cases per 100,000 people, 61 deaths, and 4,523 disability-adjusted life years (DALYs). In sensitivity analysis, strategies that included dengue vaccination were cost-effective only when the vaccine cost was 14% of the current price.

**Conclusions/Significance:**

Insecticide to prevent chikungunya and dengue in Colombia could generate significant health benefits and be cost-effective. Because of limits on diagnostic accuracy and vaccine efficacy, the cost of dengue testing and vaccination must decrease dramatically for such vaccination to be cost-effective in Colombia. The vectors for chikungunya and dengue have recently spread to new regions, highlighting the importance of understanding the effectiveness and cost-effectiveness of policies aimed at preventing these diseases.

## Author summary

Chikungunya and dengue are emerging diseases that have caused large outbreaks in various regions of the world. Both are both spread by *Aedes aegypti* and *Aedes albopictus* mosquitos. To evaluate the effectiveness and cost-effectiveness of interventions aimed at controlling either of these diseases, it is important to consider the potential effects on both diseases, as an intervention that reduces the mosquito population will reduce the spread of both diseases. We developed a dynamic transmission model of chikungunya and dengue, calibrated to data from Colombia. We evaluated the health benefits and cost-effectiveness of the following interventions: residual insecticide treatment, long-lasting insecticide-treated nets, routine dengue vaccination for children aged 9, catchup dengue vaccination for individuals aged 10–19 or 10–29, and portfolios of these interventions. In all vaccination scenarios, we considered testing for previous exposure to dengue. We found that insecticide to prevent chikungunya and dengue in Colombia could generate significant health benefits and be cost-effective. While the dengue vaccine was effective in preventing cases and deaths, costs of diagnostic testing and vaccination must decrease for dengue vaccination to be considered cost-effective.

## Introduction

Chikungunya and dengue are emerging diseases that have increasingly caused outbreaks in different regions of the world. Both viruses are spread by *Aedes aegypti* and *Aedes albopictus* mosquitos, as are Zika and yellow fever [1–3]. An estimated 50–96 million dengue infections occur worldwide each year [2,4]. Colombia experienced a major dengue outbreak in 2010 and has experienced several additional outbreaks since then [5,6]. Infection can result in dengue hemorrhagic fever and in some cases death [2]. Chikungunya has caused outbreaks in Asia, Africa, Europe, and the Americas [1,3]. Chikungunya first spread to the Americas in 2013 and caused an epidemic in Colombia in 2014 and 2015 [1,3,7,8]. Chikungunya has low mortality but high morbidity, often causing back pain and arthritis for months or years following initial infection [9].

There is no specific treatment for symptomatic chikungunya or dengue infection [2,3]. Candidate vaccines for chikungunya are in early stages of testing [10–13]. A vaccine for dengue, Dengvaxia, is publicly available [14–17], and a newer dengue vaccine candidate, TAK-003, recently completed a phase III trial [18]. However, there is evidence that Dengvaxia can increase the risk of severe infection in individuals without previous disease [19,20]. Thus, the FDA and WHO only recommend Dengvaxia for people with confirmed previous dengue infection, aged 9–16 years old (FDA) or 9–45 years old (WHO) [21,22]. However, tests to diagnose previous dengue infection are not 100% accurate. The Strategic Advisory Group of Experts (SAGE) on Immunization acknowledges that even with testing, some seronegative people may be vaccinated due to false positives [23]. Other prevention measures include insecticide, long-lasting insecticide-treated nets to cover water and windows, larvicide, and avoiding mosquito bites [3].

Previous cost-effectiveness analyses have analyzed prevention measures for chikungunya and dengue separately [24–27]. One study considered both chikungunya and dengue but focused only on larvicide and only on European cities [28]. When comparing interventions that address multiple diseases to those that do not (e.g., vector control vs. vaccination), it is useful to employ multi-disease modeling in order to include the additional health benefits of preventing cases of several diseases [29]. In this study, we use a combined chikungunya and dengue model to evaluate the cost-effectiveness of chikungunya and dengue prevention measures in Colombia.

## Methods

### Overview

**Fig 1:**
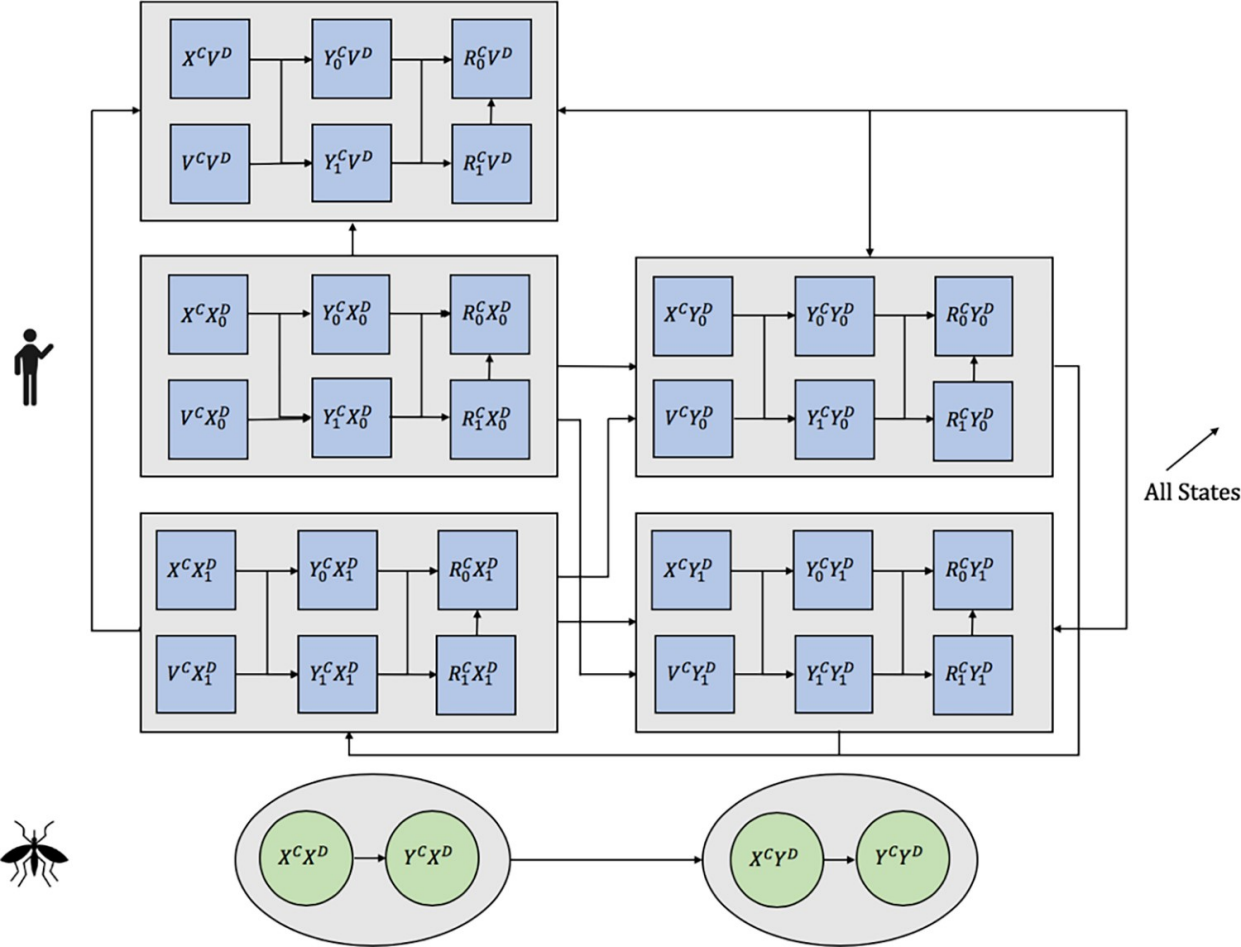
Schematic of chikungunya and dengue dynamic transmission model. *X^*i*^ = susceptible to disease *i* (*C* = chikungunya, *D* = dengue), $Y_{0}^{i}$ = infected with disease *i* with no symptoms, $Y_{1}^{i}$ = infected with disease *i* with symptoms, $R_{0}^{C}$ = recovered from chikungunya with no sequelae, $R_{1}^{C}$ = recovered from chikungunya with sequelae, V^*i*^ = vaccinated against disease *i*.

We developed a dynamic transmission model of chikungunya and dengue that considers humans and mosquitos [30]. The model captures the relevant health states for each disease and also co-infection (Fig 1). We considered residual insecticide treatment, long-lasting insecticide-treated nets used as curtains and water covers, three dengue vaccination strategies, and combinations of these interventions. We included published data in the model when available (Table 1) and calibrated the model to disease-specific incidence and deaths in Colombia from June 2014 to December 2017. We simulated results over a 5-year analytic time horizon, accounting for the full lifetime health benefits and costs of individuals alive during this period under each intervention portfolio. We calculated cost per disability-adjusted life year (DALY) averted compared to the status quo. We conducted one-way and two-way sensitivity analysis on key model variables.

**Table 1:** Parameter Values and Sources.

Parameter	Values of Best Fitting Set	Mean of Values (2^nd^, 98^th^ Percentile)	Source
**I. Epidemic parameters**			
Human birth rate per week	0.0003/person/week		[37]
Human death rate per week	0.0001/person/week		[37]
Population of Colombia in 2014	48,321,000		[41]
Probability of sequelae from symptomatic chikungunya infection	0.4757		[42]
Probability of recovering from chikungunya sequelae	0.0943*		[43]
Seroprevalence of dengue in Colombia	0.60		[44]
Probability of outpatient from symptomatic dengue ^x^	0.66		[5]
Probability of hospitalization from symptomatic dengue ^x^	0.29		[5]
Probability of dengue hemorrhagic fever from symptomatic dengue ^x^	0.05		[5]
**II. Best-fitting parameter set (mean) for uncertain epidemic parameters**			
Mosquito death rate per week	0.2919	0.2 (0, 0.8)	calibrated
Mosquito birth rate per week	0.2397	0.2 (0, 0.8)	calibrated
Initial mosquito population (millions)	18,059,000	84,404,346 (3,815,300, 190,400,000)	calibrated
Biting rate per week	2.5238	3.8 (0.7, 9.1)	calibrated
Probability of chikungunya symptoms, given infection	0.0192	0 (0, 0.1)	calibrated
Probability of chikungunya transmission to human from mosquito given bite	0.0926	0.3 (0, 1)	calibrated
Probability of chikungunya transmission to mosquito from human given bite	0.9987	0.4 (0, 1)	calibrated
Probability of chikungunya recovery in humans	0.3000	0.5 (0.3, 0.7)	calibrated
Hazard ratio of death from chikungunya	1.5064	1.8 (1.4, 2.2)	calibrated
Probability of dengue transmission to human from mosquito given bite	0.0405	0.2 (0, 1)	calibrated
Probability of dengue transmission to mosquito from human given bite	0.5040	0.3 (0, 1)	calibrated
Probability of dengue recovery in humans	0.4187	0.5 (0.3, 0.7)	calibrated
Probability of dengue symptoms given no previous infection	0.9999	0.6 (0, 1)	calibrated [45]
Probability of dengue symptoms given previous infection	0.9999	0.8 (0, 1)	calibrated
Hazard ratio of death from dengue	4.4393	5.3 (3.4, 6.8)	calibrated
Limit on the probability of infection given previous dengue infection	0.8671	0.4 (0, 1)	calibrated
Initial number of people with asymptomatic chikungunya infection	2,462	2,530.2 (17.2, 4,998.9)	calibrated
Initial number of people with symptomatic chikungunya infection	525	2,589.1 (31.6, 4,999.9)	calibrated
Initial number of mosquitos infected with chikungunya	8,850	11401.3 (686.5, 20,000)	calibrated
Initial number of people with asymptomatic dengue infection	1,066	2612.7 (0, 4,999.8)	calibrated
Initial number of people with symptomatic dengue infection	1,761	2281 (0.9, 5,000)	calibrated
Initial number of mosquitos infected with dengue	13,643	10,133.1 (440, 19,726)	calibrated
**III. Disability-Adjusted Life Years (DALYs)**			
Acute chikungunya infection	0.16–0.23		[46]
Chronic chikungunya rheumatic sequelae	0.233		[7]
Dengue infection	0.172		[46,47]
Dengue hemorrhagic fever	0.211		[46,48]
**IV. Costs**			
Healthy	$533/year		[37]
Symptomatic chikungunya direct and indirect costs	$136.81/case		[7]
Recovered no sequelae, chikungunya	$533/year		[37]
Recovered sequelae, chikungunya	$1,895.13/year		[7]
Symptomatic dengue, ambulatory, direct and indirect costs	$159.80/case^§^		[49]
Symptomatic dengue, hospitalized, direct and indirect costs	$279.56/case^§^		[49]
Dengue hemorrhagic fever, direct and indirect costs	$332.72/case^§^		[49]
Dengue vaccine cost	$20/ dose		[25]
Cost of vaccine procurement and delivery	$5.15/dose		[25]
Cost of residual insecticide treatment (RIT)	$3/household		[50,51]
Cost of dengue diagnostic test SD BIOLINE Dengue IgG/IgM	$8		[52]
Cost of dengue diagnostic test NS1 IgG ELISA	$3		[52]
Cost of long-lasting insecticide-treated net (LLIN) curtains and covers	$48/household		[36]
Average size of household	3.5		[53]
Willingness to pay (WTP, $/DALY)	$18,132^+^		[37]
**V. Intervention Efficacy and Coverage**			
Dengvaxia efficacy	0.565–0.608		[15–17]
TAK-003 dengue vaccine efficacy	0.81		[18]
Dengue seropositive test sensitivity–SD BIOLINE Dengue IgG/IgM	87.3% (84.1–90.2%)		[33]
Dengue seropositive test specificity–SD BIOLINE Dengue IgG/IgM	86.8% (83.9–89.3%)		[33]
Dengue seropositive test sensitivity–NS1 IgG ELISA	89.4%		[34]
Dengue seropositive test specificity–NS1 IgG ELISA	97.4%		[34]
Residual insecticide treatment (RIT) efficacy	63.9%		[35]
Long-lasting insecticide-treated net (LLIN) curtains and covers efficacy	71%		[36]
Percent of total population 9 years old	1.79%		[54]
Percent of total population 10–19 years old	18.19%		[54]
Percent of total population 10–29 years old	35.30%		[54]

*Calculated from mean total clinical duration, infection and sequelae = 10 weeks

^x^ Mean of total dengue cases from 2010–2012 in Colombia

^§^ 2012 dollars, adjusted for inflation [40]

^+^ WTP based on 3 times the GDP per capita in Colombia in 2015 ($6044)

### Model structure

In the chikungunya natural disease history model, humans and mosquitos are born susceptible. People are infected with chikungunya at a rate that depends on the probability of transmission, the biting rate, the size of the infected mosquito population, and the fraction of people who are susceptible. People infected with chikungunya may develop symptoms and have a higher probability of mortality during symptomatic infection [31]. People may also develop long-term sequelae after recovering from a symptomatic chikungunya infection but not after an asymptomatic infection. Recovery from chikungunya results in complete immunity against future infections. People are vaccinated against chikungunya continuously for the routine vaccination and at the beginning of the analytic time horizon for the catchup vaccine. Only individuals who are susceptible to chikungunya are vaccinated because vaccination has no effect in infected or previously infected individuals. Mosquitos are infected with chikungunya at a rate depending on the probability of transmission from humans to mosquitos, the biting rate, the size of the infected human population (symptomatic and asymptomatic), and the fraction of mosquitos that are susceptible. Mosquitos do not recover from chikungunya in the model.

In the dengue disease history model, humans are born susceptible with no previous infection, and mosquitos are born susceptible. People who are susceptible with no previous infection history can become infected with the force of infection that depends on the probability of transmission from mosquitos to humans, the biting rate, the size of the infected mosquito population, and the fraction of people who are susceptible. After an infection, people recover and become susceptible again but now have history of previous infection. Since dengue has four serotypes, infection only results in immunity to a single serotype [2,32], repeated dengue infections are possible, but decrease in probability after initial infection. The probability of infection is lower for susceptible individuals with previous infection than for susceptible individuals with no previous infection. Notably, repeated infection is more likely to result in symptomatic dengue. Of the total number of people with symptomatic infection, we assumed that 66% are outpatients, 29% are hospitalized, and 5% are hospitalized with dengue hemorrhagic fever (DHF) [5]. People with a symptomatic infection have a higher risk of death. Mosquitos are infected with dengue at a rate that depends on the probability of transmission from humans to mosquitos, the biting rate, the size of the infected human population (symptomatic and asymptomatic), and the fraction of mosquitos that are susceptible. Mosquitos do not recover from dengue.

We assumed that co-infection does not affect transition probabilities and health outcomes or costs incurred due to each disease are added during coinfection. The probability of infection for both diseases depends on the same mosquito biting rate but there are different probabilities of transmission to humans from mosquitos and vice versa, given a bite. We used a deterministic cohort model of Colombia’s population. Modeling was performed using ordinary differential equations (Equation A in S1 Text) in MATLAB. We modeled the chikungunya and dengue epidemics from June 2014 to June 2019 and followed cohorts for a lifetime to calculate health effects and costs.

### Calibration

Using directed search, we calibrated 22 uncertain model parameters (Table A in S1 Text) to weekly Colombia data on incidence, cumulative incidence, and deaths from June 2014 to December 2017. We calibrated symptomatic cases only as these were observed and contained in the empirical data, although both symptomatic and asymptomatic cases contributed to infection dynamics. After drawing initial parameters from distributions, we minimized the weighted goodness of fit error (Equation B in S1 Text) by varying these parameters to calculate the optimized parameter set. We repeated this process for 2160 parameter sets. For our analyses, we considered the 300 best-fitting parameter sets.

### Interventions

We considered interventions that include residual insecticide treatment, long-lasting insecticide-treated nets used as curtains and water covers, three dengue vaccination strategies, and combinations of these interventions. We compared the interventions with the status quo policy of local government programs in Colombia that included surveillance, educational prevention campaigns, and vector control [5]. We assumed intervention efficacy based on existing studies (Table 1).

In the base case, we assumed the dengue vaccine would be similar to Dengvaxia. To model vaccination, we created a separate state for the susceptible, vaccinated portion of the population (Fig 1). We assumed that the vaccine reduces the probability of transmission from mosquitos to humans for vaccinated individuals by 60%, based on efficacy data from clinical trials [15–17,24]. No infected individuals are considered for dengue vaccination. All dengue vaccinated individuals have a probability of symptoms and death upon infection equal to those of a secondary infection but a lower probability of infection. Because Dengvaxia is a tetravalent vaccine, we assumed that 40% of individuals who become infected with dengue after vaccination no longer have any protection from the vaccine and 60% still have protection. We assumed the best-case scenario for vaccine use: the vaccine has constant efficacy at the full level found in clinical trials and no loss to follow-up occurs during the three-dose vaccination. The coverage rate refers to the number of people who are tested for previous dengue exposure with intention to vaccinate.

We assume that the sensitivity and specificity of the dengue diagnostic test is the same as that of the SD BIOLINE Dengue IgG/IgM test, a rapid test used to assess previous infection that can be used in the field [33]; thus we assumed 87.3% sensitivity and 86.8% specificity. Although the NS1 IgG ELISA test is more accurate [34], it must be conducted in a laboratory and would not be ideal for a mass vaccination campaign. Individuals with a true positive test (i.e., those with previous exposure to dengue) are vaccinated, as are individuals with a false positive test (i.e., with no previous dengue exposure but with a positive test). We included the costs of diagnostic tests but not the costs of administering diagnostic tests.

We considered three vaccination strategies: routine vaccination, a continuous vaccination campaign for all children 9 years old; a one-time catchup vaccination campaign for all individuals aged 10–19; and a one-time catchup vaccination campaign for all individuals aged 10–29. Routine vaccination was constant throughout the analytic time horizon, with all individuals tested before vaccination. Catchup vaccination was modeled as a one-time vaccination at the beginning of the analytic time horizon. The total cost of dengue vaccination included the cost of diagnostic tests for everyone tested plus the cost of vaccination and administrative costs for all vaccinated individuals.

We assumed that residual insecticide treatment is applied four times per year per household and that it decreases the initial mosquito population and increases the mosquito mortality rate by 63.9% continuously [35]. We assumed that long-lasting insecticide-treated nets (LLIN) decrease the mosquito birth rate by 71% continuously and are replaced annually [36]. We assumed that these interventions are implemented at the beginning of the modeled time period with a coverage rate of 5% of the human population.

### Cost-effectiveness analysis

We calculated DALYs and costs in 2015 US dollars for all health states for the lifetime of the cohort. We discounted DALYs and costs at 3% annually. We compared incremental cost per DALY averted to willingness-to-pay (WTP) thresholds based on 2015 GDP per capita of Colombia [37] for cost-effective and very cost-effective interventions ($18,132 and $6,044 per DALY averted, respectively). We assessed the preferred strategy by selecting the intervention with the largest incremental cost per DALY averted less than $18,132.

### Sensitivity analysis

We conducted one-way sensitivity analysis on intervention costs and efficacy, intervention coverage, diagnostic accuracy, DALY values, discount factor, and analytic time horizon. We performed two-way sensitivity analysis on vaccine and diagnostic costs, vaccine costs and efficacy, and vaccine efficacy and diagnostic accuracy.

### Scenario analyses

Chikungunya vaccine candidates are still in early stages of development and their efficacy in humans is uncertain. We conducted a scenario analysis to assess whether a chikungunya vaccinate strategy, with no test, would be cost-effective. In addition, although it would be operationally more difficult, we considered the DENV Detect IgG ELISA dengue diagnostic test, instead of the rapid test. For this analysis, we included the cost of the test, but did not include additional laboratory and transportation costs that might be incurred. We also considered the TAK-003 dengue vaccine, which is not yet on the market, assuming 81% efficacy for two doses. We assessed the same testing and vaccination strategies to find the price threshold at which this vaccine would be cost-effective for Colombia. Finally, we considered a scenario with more expensive and less effective vector control and less expensive and more effective dengue vaccination: specifically, we considered a scenario in which insecticide and LLIN costs increased threefold and efficacy was halved while vaccine efficacy increased to 90% and vaccine and diagnostic costs decreased to one-third of the base case.

## Results

### Calibration

Calibration produced 300 feasible parameter sets that reflected the incidence of and deaths from chikungunya and dengue in Colombia over the period June 2014 to December 2017 (Fig A in S1 Text). Parameter distributions after calibration differed from the initial distributions (Fig B in S1 Text). Correlations between parameters show the interdependency and constraints of the dynamic transmission model (Fig C in S1 Text). For example, the mosquito death rate is positively correlated with the mosquito birth rate, as the target mosquito population size is stable.

### Health effects

**Fig 2:**
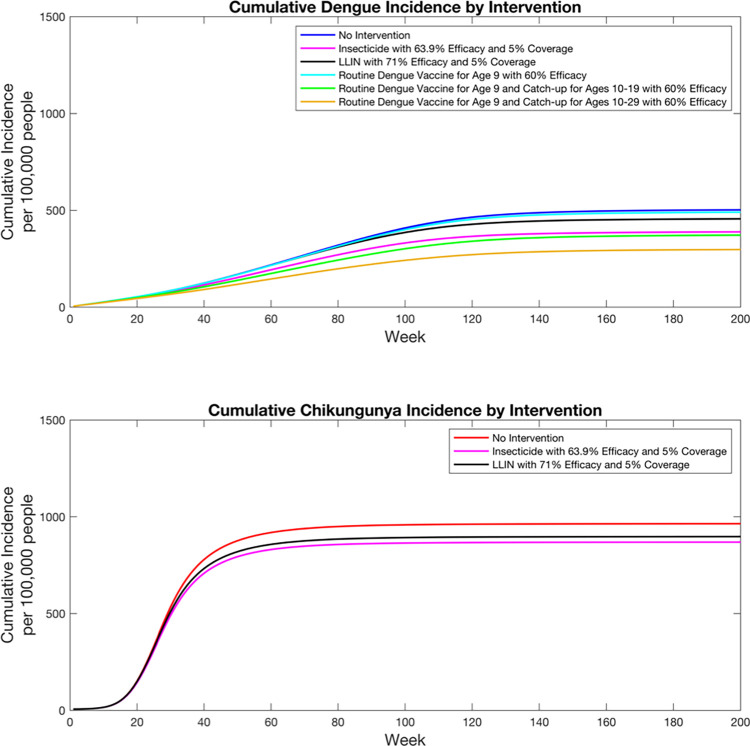
Expected cumulative incidence by disease and intervention.

**Fig 3:**
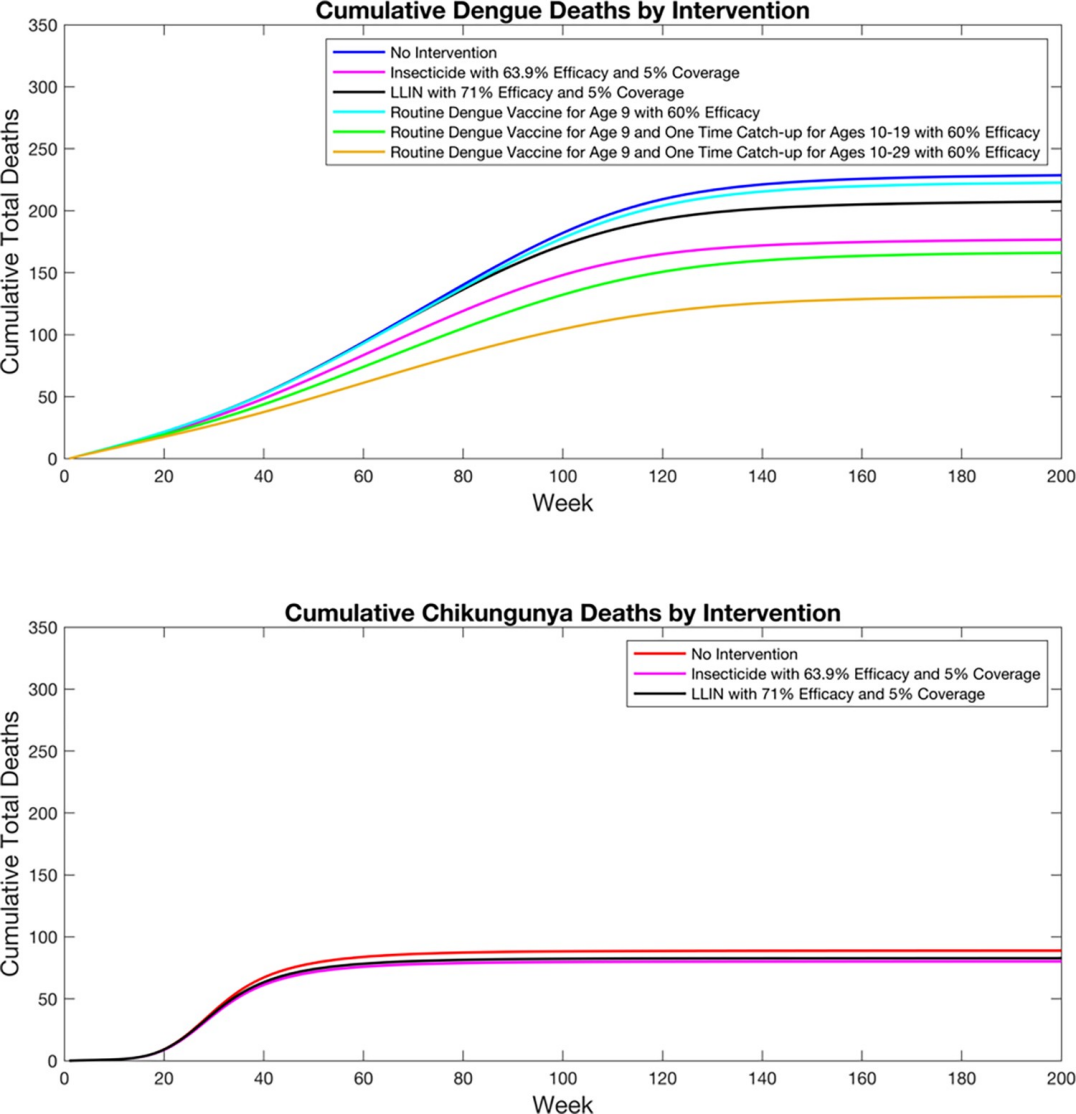
Expected cumulative deaths by disease and intervention.

Mean cumulative incidence and deaths for chikungunya and dengue during the analytic time horizon are shown in Figs 2 and 3 and Table 2. Minimum and maximum cumulative incidence and deaths for chikungunya and dengue during the time horizon for each intervention are shown in Figs D-G in S1 Text. DALYs averted are shown in Table 3. Insecticide averted an estimated 95 chikungunya cases and 114 dengue cases per 100,000 people, 61 deaths, and 4,523 DALYs. LLIN averted an estimated 67 chikungunya cases and 46 dengue cases per 100,000 people, 27 deaths, and 2,404 DALYs. Routine dengue vaccination at age 9 averted an estimated 0 chikungunya cases, 12 dengue cases per 100,000 people, 6 deaths, and 301 DALYs. Routine dengue vaccination at age 9 plus catchup vaccination for individuals aged 10–19 averted an estimated 0 chikungunya cases, 132 dengue cases per 100,000 people, 63 deaths, and 3,375 DALYs. Routine dengue vaccination at age 9 plus catchup vaccination for individuals aged 10–29 averted an estimated 0 chikungunya cases, 207 dengue cases per 100,000 people, 89 deaths, and 5,355 DALYs. The portfolio with insecticide, LLIN, and routine dengue vaccination at age 9 with catchup vaccination for individuals aged 10–29 had the largest health effects, averting an estimated 151 chikungunya cases and 261 dengue cases per 100,000 people, 137 deaths, and 9,628 DALYs.

**Table 2:** Expected Health Outcomes (2^nd^, 98^th^ Percentile) for Each Strategy and Comparison to Status Quo.

Intervention	Cumulative Chikungunya Incidence/ 100,000 people	Cumulative Dengue Incidence/ 100,000 people	Cumulative Chikungunya Cases Averted/ 100,000 people	Cumulative Dengue Cases Averted/ 100,000 people	Chikungunya Deaths	Dengue Deaths	Total Deaths	Deaths Averted
Status Quo	965 (961, 995)	505 (476, 594)	-	-	89 (79, 93)	230 (215, 286)	319 (296, 376)	-
Insecticide	869 (587, 994)	391 (207, 587)	95 (1, 380)	114 (16, 292)	80 (52, 92)	178 (94, 270)	258 (151, 362)	61 (7, 166)
LLIN	898 (603, 995)	459 (278, 594)	67 (0, 361)	46 (0, 230)	83 (54, 92)	209 (126, 272)	291 (190, 363)	27 (0, 126)
Routine Dengue Vaccination	965 (961, 995)	492 (447, 592)	0 (0, 0)	12 (3, 51)	89 (79, 93)	224 (199, 277)	313 (287, 367)	6 (1, 24)
Routine Dengue Vaccination + Catchup for Ages 10–19	965 (961, 995)	372 (113, 558)	0 (0, 0)	132 (25, 381)	89 (79, 93)	167 (50, 259)	256 (133, 347)	63 (12, 176)
Routine Dengue Vaccination + Catchup for Ages 10–29	965 (961, 995)	298 (44, 536)	0 (0, 0)	207 (47, 449)	89 (79, 93)	131 (19, 235)	220 (108, 327)	98 (21, 207)
Insecticide + Routine Dengue Vaccination	869 (587, 994)	384 (195, 572)	95 (1, 380)	121 (25, 305)	80 (52, 92)	174 (88, 269)	254 (145, 361)	64 (12, 172)
Insecticide + Routine Dengue Vaccination + Catchup for Ages 10–19	869 (587, 994)	304 (72, 549)	95 (1, 380)	201 (49, 420)	80 (52, 92)	136 (32, 251)	216 (89, 340)	103 (25, 228)
Insecticide + Routine Dengue Vaccination + Catchup for Ages 10–29	869 (587, 994)	250 (36, 533)	95 (1, 380)	255 (70, 457)	80 (52, 92)	110 (15, 229)	190 (72, 321)	129 (37, 245)
LLIN + Routine Dengue Vaccination	898 (603, 995)	450 (273, 582)	67 (0, 361)	55 (3, 237)	83 (54, 92)	204 (124, 270)	287 (188, 362)	32 (1, 128)
LLIN + Routine Dengue Vaccination + Catchup for Ages 10–19	898 (603, 995)	351 (103, 548)	67 (0, 361)	154 (33, 390)	83 (54, 92)	157 (46, 259)	240 (104, 347)	79 (16, 211)
LLIN + Routine Dengue Vaccination + Catchup for Ages 10–29	898 (603, 995)	285 (44, 533)	67 (0, 361)	220 (58, 449)	83 (54, 92)	125 (19, 235)	208 (77, 327)	110 (28, 238)
Insecticide + LLIN	814 (361, 994)	371 (145, 566)	151 (1, 601)	133 (16, 361)	75 (33, 92)	169 (65, 270)	244 (103, 362)	75 (7, 220)
Insecticide + LLIN + Routine Dengue Vaccination	814 (361, 994)	365 (142, 562)	151 (1, 601)	139 (25, 364)	75 (33, 92)	166 (64, 269)	241 (102, 361)	78 (13, 222)
Insecticide + LLIN + Routine Dengue Vaccination + Catchup for Ages 10–19	814 (361, 994)	293 (69, 546)	151 (1, 601)	211 (55, 433)	75 (33, 92)	131 (31, 251)	206 (66, 339)	113 (28, 249)
Insecticide + LLIN + Routine Dengue Vaccination + Catchup for Ages 10–29	814 (361, 994)	243 (36, 531)	151 (1, 601)	261 (77, 457)	75 (33, 92)	107 (15, 229)	182 (53, 321)	137 (41, 263)

LLIN = long-lasting insecticide-treated nets; Routine Dengue Vaccination corresponds to vaccination of all 9-year-olds.

**Table 3:** Expected Intervention Costs, DALYs, and Net Monetary Benefit (2^nd^, 98^th^ Percentile) and Cost per DALY Averted for Each Strategy.

Intervention	Total Cost (2015 USD, millions)	Incremental Costs (2015 USD, millions)	Total DALYs	DALYs Averted	NMB (2015 USD)	ICER (2015 USD)
Status Quo	751,627 (751,620, 751,645)	--	77,103,196 (77,094,183, 77,111,793)	--	--	--
Insecticide	751,642 (751,583, 751,684)	15 (-42, 38)	77,098,673 (77,086,087, 77,110,113)	4523 (353, 14,123)	67 (-31, 296)	3279
LLIN	751,771 (751,709, 751,802)	143 (83, 158)	77,100,793 (77,087,566, 77,111,176)	2404 (0, 11,588)	-100 (-158, 127)	59,647
Routine Dengue Vaccination	751,841 (751,825, 751,934)	213 (198, 288)	77,102,895 (77,093,954, 77,111,656)	301 (66, 1143)	-208 (-283, -179)	708,211
Routine Dengue Vaccination + Catchup for Ages 10–19	752,265 (752,178, 752,363)	638 (545, 728)	77,099,821 (77,090,695, 77,109,931)	3375 (846, 8824)	-576 (-701, -443)	188,887
Routine Dengue Vaccination + Catchup for Ages 10–29	752,667 (752,456, 752,781)	1040 (823, 1144)	77,097,842 (77,088,414, 77,108,518)	5355 (1563, 10,517)	-943 (-1097, -766)	194,208
Insecticide + Routine Dengue Vaccination	751,856 (751,788, 751,971)	228 (162, 326)	77,098,491 (77,085,984, 77,109,922)	4705 (597, 14,416)	-143 (-314, 98)	48,529
Insecticide + Routine Dengue Vaccination + Catchup for Ages 10–19	752,284 (752,212, 752,401)	657 (585, 765)	77,096,344 (77,084,133, 77,108,540)	6853 (1429, 16,877)	-532 (-731, -279)	95,826
Insecticide + Routine Dengue Vaccination + Catchup for Ages 10–29	752,689 (752,502, 752,818)	1061 (869, 1181)	77,094,841 (77,082,608, 77,107,573)	8356 (2223, 18,081)	-910 (-1124, -666)	127,015
LLIN + Routine Dengue Vaccination	751,984 (751,916, 752,091)	357 (289, 445)	77,100,568 (77,087,394, 77,110,957)	2628 (71, 11,760)	-309 (-440, -76)	135,806
LLIN + Routine Dengue Vaccination + Catchup for Ages 10–19	752,411 (752,334, 752,521)	783 (701, 885)	77,097,977 (77,084,761, 77,109,253)	5220 (915, 15,900)	-689 (-857, -417)	150,063
LLIN + Routine Dengue Vaccination + Catchup for Ages 10–29	752,814 (752,612, 752,937)	1187 (979, 1301)	77,096,198 (77,083,017, 77,108,012)	6999 (1594, 17,536)	-1060 (-1252, -795)	169,563
Insecticide + LLIN	751,790 (751,702, 751,841)	162 (76, 195)	77,097,100 (77,080,890, 77,110,008)	6097 (356, 20,404)	-52 (-189, 289)	26,608
Insecticide + LLIN + Routine Dengue Vaccination	752,003 (751,906, 752,129)	376 (280, 483)	77,096,944 (77,080,822, 77,109,819)	6253 (644, 20,466)	-262 (-472, 86)	60,075
Insecticide + LLIN + Routine Dengue Vaccination + Catchup for Ages 10–19	752,433 (752,335, 752,558)	805 (708, 923)	77,094,985 (77,079,376, 77,108,448)	8211 (1605, 21,716)	-656 (-888, -326)	98,052
Insecticide + LLIN + Routine Dengue Vaccination + Catchup for Ages 10–29	752,838 (752,662, 752,975)	1210 (1032, 1339)	77,093,568 (77,078,360, 77,107,195)	9628 (2386, 22,940)	-1036 (-1280, -693)	125,699

LLIN = long-lasting insecticide-treated nets; NMB = Net monetary benefit, calculated assuming a willingness to pay of $18,132 per DALY; ICER = incremental cost per DALY averted, compared to status quo; Routine Dengue Vaccination corresponds to vaccination of all 9-year-olds.

### Cost-effectiveness analysis

**Fig 4:**
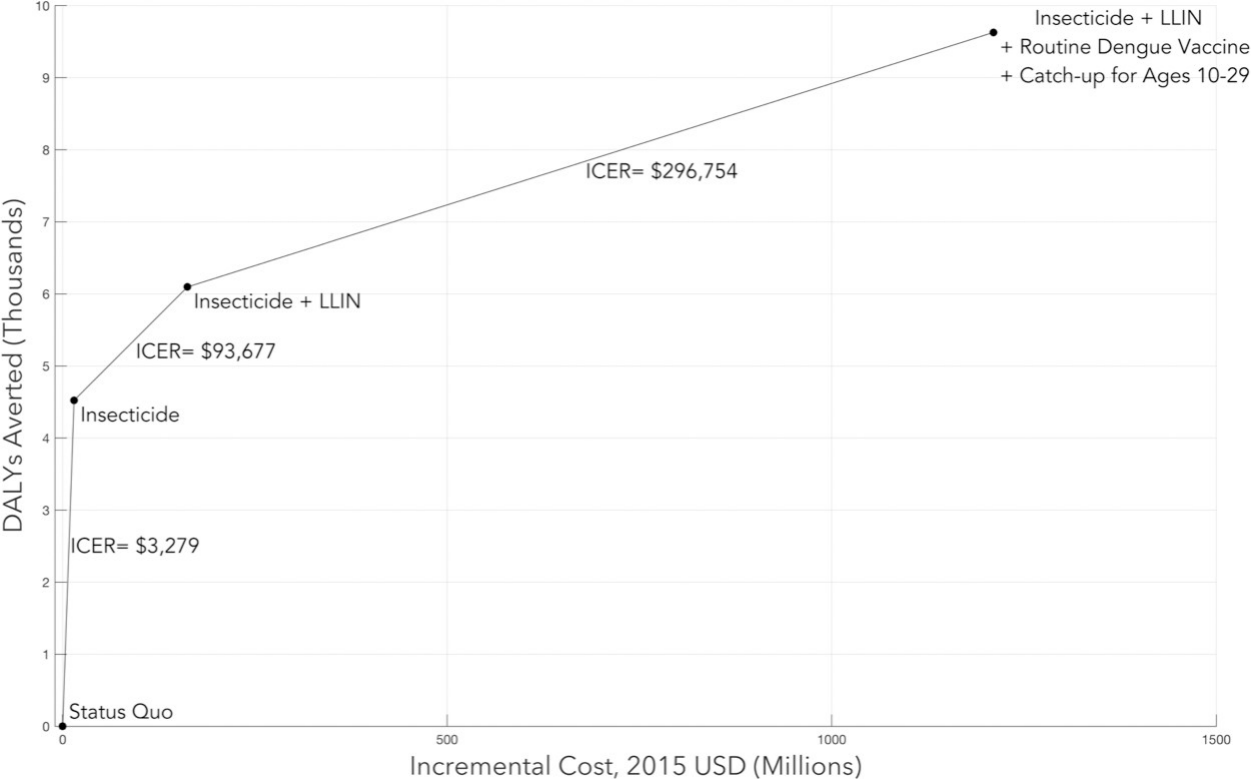
Incremental costs, DALYs averted, and incremental cost-effectiveness ratio for strategies on the cost-effectiveness frontier. LLIN = long-lasting insecticide-treated net.

Table 3 shows expected incremental costs and benefits for all strategies compared to the status quo and Fig 4 shows the efficient frontier. Insecticide is cost-effective and is the preferred strategy with an incremental cost-effectiveness ratio of $3,279/ DALY averted. The next strategy on the frontier includes insecticide along with LLIN. The incremental cost-effectiveness ratio of this strategy compared to insecticide alone is $93,677 per DALY averted. Since the GDP per capita in Colombia is approximately $6,044, this strategy would not be considered cost-effective. The other strategy on the frontier, the portfolio including LLIN, insecticide, and dengue vaccination with catchup ages 10–29, costs even more per DALY averted and is not cost-effective.

**Fig 5:**
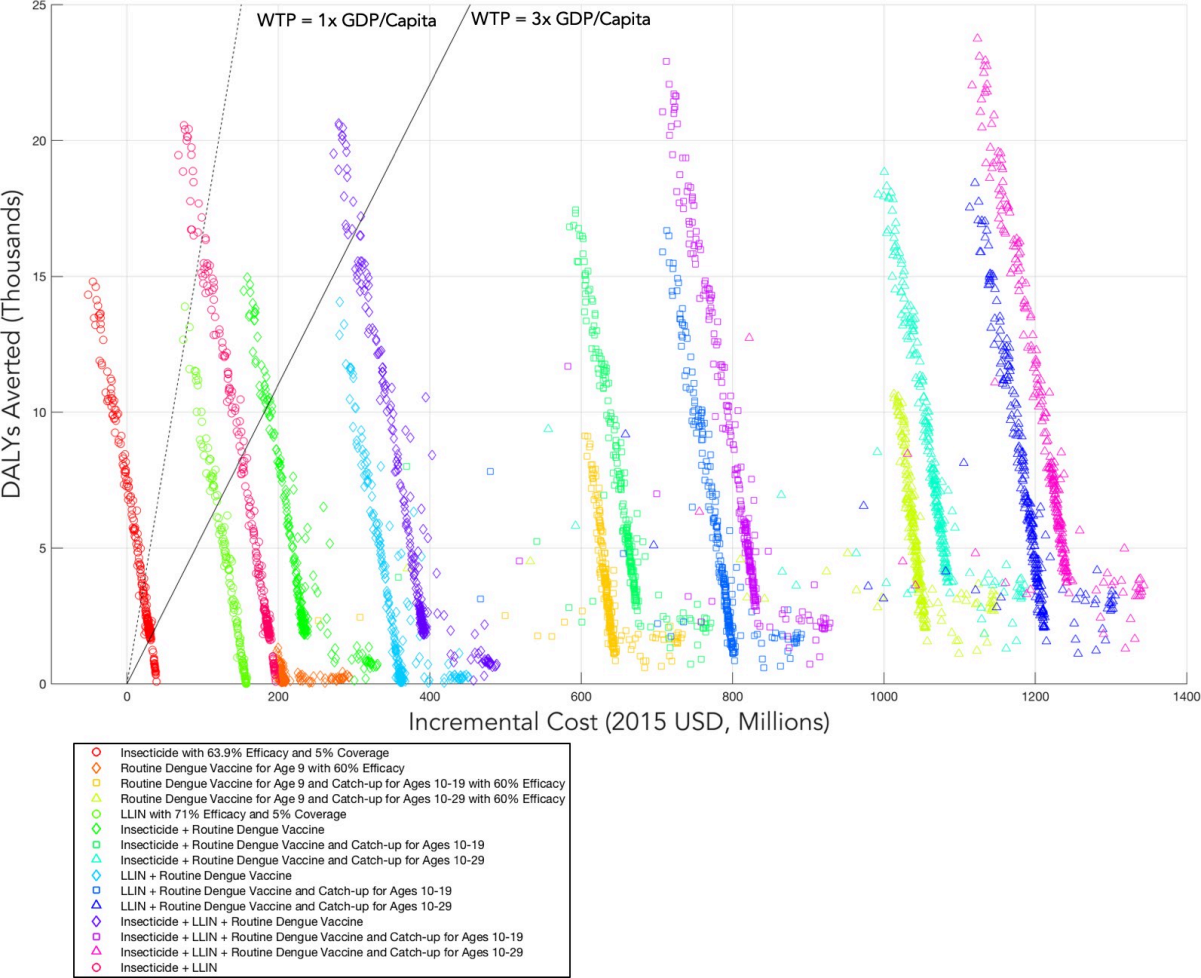
Incremental costs and DALYs averted for all strategies and all parameter sets.

Fig 5 shows costs and DALYs averted for each of the 300 parameter sets for each of the strategies we considered. For our base case assumptions at a willingness to pay of $18,132, insecticide was the preferred strategy in 86% of cases over the 300 parameter sets, insecticide plus LLIN was preferred in 1% of cases, and the status quo was preferred in 13% of cases (Table B in S1 Text, first column).

### Sensitivity analysis

The preferred strategies remained unchanged when we considered 100% vaccine efficacy, a no-cost dengue vaccine, or 100% diagnostic sensitivity and specificity or no cost for the dengue diagnostic test. In two-way sensitivity analysis, the preferred strategies were unchanged with 100% sensitivity and specificity for the dengue vaccine and 100% vaccine efficacy. Even with 100% vaccine efficacy and $0 vaccine cost and $8 diagnostic test cost, dengue vaccination was preferred to insecticide in only one parameter set.

**Fig 6:**
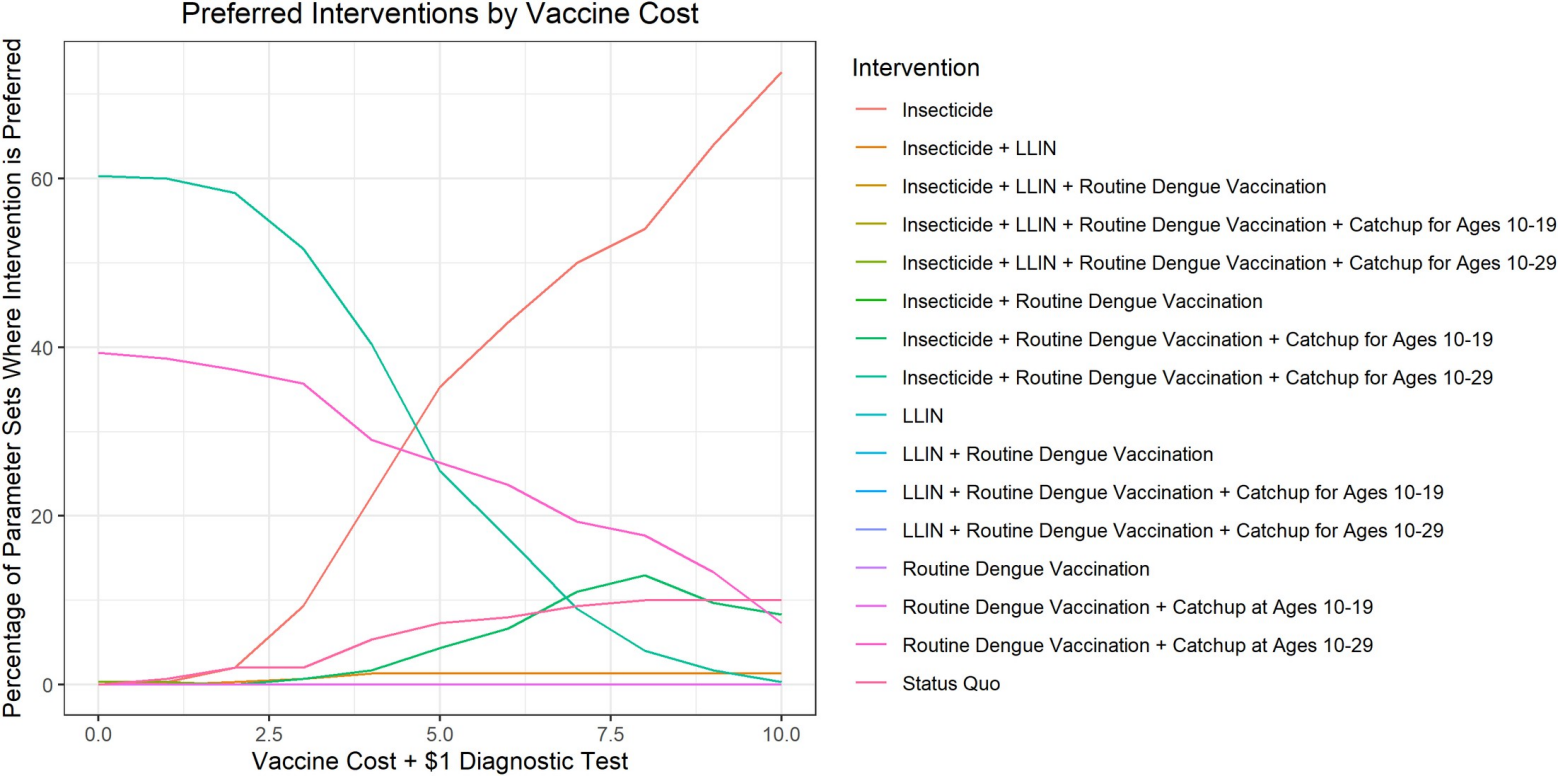
Sensitivity analysis: Preferred intervention by cost of vaccine when diagnostic test cost is set to $1.

The base case analysis assumed $8 for the dengue diagnostic test and $75.45 for dengue vaccination. In sensitivity analysis we varied these costs down to $0 (Fig 6, Table B in S1 Text). The threshold for interventions including dengue vaccination to be preferred in a majority of the parameter sets was $5 for vaccination cost and $1 for diagnostic costs. The threshold for a Dengvaxia intervention to be cost-effective compared to the status quo was $9.75 for the three-dose vaccine cost and $1 for diagnostic costs for routine dengue vaccination with catchup for ages 10–29. Thus, unless the cost of a dengue vaccine is a fraction of the current cost of Dengvaxia and diagnostic testing cost is only a small fraction of SD BIOLINE Dengue IgG/IgM cost, dengue vaccination in Colombia is unlikely to be cost-effective.

The decision was sensitive to the efficacy and cost of insecticide and cost of LLIN (Table C in S1 Text). Insecticide efficacy must be above 20% in order to be cost-effective compared to the status quo. In one-way sensitivity analysis, insecticide was cost-effective for coverage levels of 1%-14% and costs under $9 per household. In one-way sensitivity analysis of LLIN efficacy, coverage, and cost, we found that LLIN is cost-effective compared to the status quo if the cost is less than $19 per household; the result is not sensitive to efficacy or coverage.

### Scenario analyses

In a scenario analysis of a hypothetical chikungunya vaccine, the chikungunya vaccination strategy was not cost-effective (Table D in S1 Text). When using the DENV Detect IgG ELISA dengue diagnostic test before Dengvaxia, the vaccination strategies are not cost-effective (Table E in S1 Text). Because the test’s specificity is higher than that of the SD BIOLINE Dengue IgG/IgM test, the intervention vaccinates fewer individuals who have not been exposed to dengue. Although this decreases the probability of severe symptoms and death, it also decreases the total number of vaccinations, resulting in lower costs and fewer DALYs averted. We also considered the newer TAK-003 vaccine and found that it is more effective and less costly than Dengvaxia due to the higher efficacy and two-dose strategy (Table F in S1 Text), but is still not cost-effective. The threshold for TAK-003 to be the preferred strategy is $6 for a two-dose vaccine cost and $1 for diagnostic costs. Both vaccine plus catchup strategies are cost-effective compared to the status quo under $9.50 per two-dose vaccine and $1 per diagnostic test.

When comparing a more effective and less expensive dengue vaccine to less effective and more expensive insecticide and LLIN, we found that insecticide was the only intervention that was cost-effective compared to the status quo (Table G in S1 Text). The status quo was preferred in 84% of cases of the 300 parameter sets and insecticide was preferred in 16% of cases.

## Conclusions

Chikungunya and dengue cause significant morbidity and mortality in Colombia and elsewhere. We found that residual insecticide treatment with 63.9% efficacy and 5% coverage is likely to be cost-effective in Colombia, generating significant health benefits at relatively low cost. Unless the prices of dengue vaccination and diagnostic tests decrease significantly, such vaccination is unlikely to be cost-effective in Colombia. Diagnostic testing accounts for a significant portion of total vaccination intervention cost because many individuals who are tested are not vaccinated. However, including testing to prevent vaccine-caused symptoms or death is important to maintain trust in vaccination campaigns for all diseases.

Our cost-effectiveness analysis is novel in developing a joint chikungunya and dengue model that uses a common mosquito population. Our use of a combined dengue and chikungunya model allowed us to capture more of the health benefits of the vector-control interventions like insecticide and LLIN because it included the benefits of preventing both diseases while spending the same amount on implementation costs [29]. A multi-disease model also allows for better estimation of coinfection of chikungunya and dengue and considers competing mortality; both viruses were widespread from 2014–2017 in Colombia. While chikungunya and dengue had been considered together previously in a non-endemic area in Europe [28], we modeled both with a dynamic infectious disease model with a common mosquito population in an endemic area, and with more interventions including vaccination and insecticide.

Our analysis has several limitations. We did not consider the four serotypes of dengue separately but instead assumed an aggregated accuracy for the dengue vaccine, although there are differences in efficacy according to serotype. Since the four serotypes of dengue are prevalent in Colombia, we modeled the serotypes as repeating infections. This method would not be appropriate in countries where only one serotype is prevalent or for studies that focus on the separate dynamics of dengue serotypes. We did not include age stratification in our model as we did not have data on the age-specific probability of infection. We instead assumed that vaccination applied to a percentage of the total population and used the model to show the effect on transmission dynamics if a certain percentage of the population is vaccinated. We assumed no loss to followup in vaccination campaigns, no decrease in vaccine efficacy over time, and no cost of diagnostic test administration. Including loss to followup between the multiple doses would decrease the effectiveness of the vaccine and make it even less cost-effective than we estimated, and including administrative costs for diagnostic tests would make vaccination campaigns even more costly. We did not include the additional benefits of insecticide in preventing Zika and yellow fever. Inclusion of these additional benefits would make insecticide appear even more favorable. We calibrated our models to incidence and death data on suspected and confirmed cases, which could be underestimates of symptomatic cases. We did not consider geographic heterogeneity in the infectious disease model, as another study has done [38]. Our analysis focuses on Colombia during large outbreaks of chikungunya and high incidence of dengue. Lower prevalence of both diseases during the analytic time periods would most likely make all interventions less effective.

Our research shows that in the context of Colombia, where both chikungunya and dengue are present, it is important to consider interventions that can prevent multiple mosquito-borne diseases. Our analysis showed insecticide to be cost-effective because it has low cost and prevents multiple diseases. We showed that vaccination could be cost-effective with a decrease in testing and vaccination costs, as vaccination was the most effective intervention. While we found that insecticide was generally more cost-effective than vaccination, our analysis did not include potential negative effects of insecticide, constraints on the amount of insecticide that could be sprayed by professionals in Colombia, or insecticide resistance. Our study also did not consider how these interventions would fit into a national budget, how they could be implemented in Colombia, or how effective each intervention would be in addressing local outbreaks. Future research is needed to assess these factors.

Future research should be also carried out using multi-disease models to study the cost-effectiveness of dengue and chikungunya prevention strategies in other countries. Due to variability in the timing of outbreaks, the prevalence of dengue serotypes, and the prevalence of mosquitos, different geographic regions can have different epidemic characteristics. It would be important to distinguish how different epidemic characteristics affect the cost-effectiveness of control programs. The example of Colombia provides insights for interventions in countries with endemic levels of dengue during a chikungunya outbreak or countries with large non-exposed populations where outbreaks are expected to occur.

The vectors for chikungunya and dengue have recently spread to new geographic areas around the world, including the United States [32,39], so it is important to understand the effectiveness and cost-effectiveness of policies aimed at preventing these diseases. Models of the type we have developed can be useful in informing decisions regarding chikungunya and dengue control in Colombia and elsewhere.

## Supporting information

S1 Text Supplement–prevention and control of Chikungunya and DENGUE in Colombia: A cost-effectiveness analysis. **Equation A.** Differential Equations to Model Chikungunya and Dengue Transmission Dynamics (adapted from Keeling and Rohani’s mosquito-borne disease SIR model. **Equation B**. Goodness of Fit Error (GOF) of Model Outputs For Target Data *i* at Time *t* over Time Period 1 to *τ* weeks. **Table A. Distributions of Initial Parameter Sets for Calibration. Table B. Percent of Parameter Sets that Prefer Each Intervention, by Vaccine Cost (C**_**V**_**) and Diagnostic Test Cost (C**_**D**_**).** Preferred strategy is defined as the intervention with the minimum incremental cost/DALY averted less than $18,132. If each incremental cost/DALY averted is greater than the WTP, the status quo is preferred; LLIN = long-lasting insecticide-treated nets; Routine Dengue Vaccination corresponds to vaccination of all 9-year-olds. * Base case. **Table C. One-way Sensitivity Analysis of Insecticide and LLIN Efficacy, Cost, and Coverage.** LLIN = long-lasting insecticide-treated nets. **Table D. Scenario Analysis of Hypothetical Chikungunya Vaccine**. NMB = Net monetary benefit, calculated assuming a willingness to pay of $18,132 per DALY; ICER = incremental cost per DALY averted, compared to status quo; Routine Dengue Vaccination corresponds to vaccination of all 9-year-olds. **Table E. Scenario Analysis of Test and Vaccinate Strategy using the DENV Detect IgG ELISA Dengue Diagnostic Test and Dengvaxia.** LLIN = long-lasting insecticide-treated nets; Routine Dengue Vaccination corresponds to vaccination of all 9-year-olds; NMB = Net monetary benefit, calculated assuming a willingness to pay of $18,132 per DALY; ICER = incremental cost per DALY averted, compared to status quo. **Table F. Scenario Analysis of Test and Vaccinate Strategy with TAK-003 Dengue Vaccine.** LLIN = long-lasting insecticide-treated nets; Routine Dengue Vaccination corresponds to vaccination of all 9-year-olds; NMB = Net monetary benefit, calculated assuming a willingness to pay of $18,132 per DALY; ICER = incremental cost per DALY averted, compared to status quo. Table G. Scenario Analysis of More Effective, Less Costly Vaccine and Less Effective, More Costly Insecticide and LLIN*. *Vaccine efficacy = 90%; Vaccine and diagnostic costs at one-third of base case; Insecticide and LLIN costs triple base case; Insecticide and LLIN efficacy half of base case; LLIN = long-lasting insecticide-treated nets; Routine Dengue Vaccination corresponds to vaccination of all 9-year-olds; NMB = Net monetary benefit, calculated assuming a willingness to pay of $18,132 per DALY; ICER = incremental cost per DALY averted, compared to status quo. **Fig A. Calibration results: 300 best-fitting parameter sets. Fig B. Distributions of model parameter values before and after calibration.** Blue denotes distribution for each parameter before calibration, orange denotes distribution of each parameter after calibration. Fig C. Parameter correlation matrix for the 300 best-fitting parameter sets*. *One-to-one correlations were found for mosquito birth rate/mosquito death rate, dengue death hazard/probability of recovery, and chikungunya death hazard/probability of chikungunya recovery. These come from the necessary steady state needed for each parameter. For example, an increase in the mosquito birth rate also means an increase in mosquito deaths to keep a stable population. An increase in the death hazard during infection is also correlated with an increase in the probability of recovery (which decreases the time an individual is susceptible for higher mortality hazard). **Fig D. Cumulative incidence of dengue by intervention in Colombia: June 2014 to December 2017.** Solid line denotes the mean, and dashed lines denote the range. **Fig E. Cumulative incidence of chikungunya by intervention in Colombia: June 2014 to December 2017.** Solid line denotes the mean, and dashed lines denote the range. **Fig F. Cumulative dengue deaths by intervention in Colombia: June 2014 to December 2017** Solid line denotes the mean, and dashed lines denote the range. **Fig G. Cumulative chikungunya deaths by intervention in Colombia: June 2014 to December 2017.** Solid line denotes the mean, and dashed lines denote the range.
(DOCX)
