# Supplementary material for: Prevention and control of dengue and chikungunya in Colombia: A cost-effectiveness analysis
Source: PLoS Negl Trop Dis. 2021 Dec 29;15(12):e0010086. doi: 10.1371/journal.pntd.0010086 (PMC8752007; doi:10.1371/journal.pntd.0010086)
Supplement: S1 Text — Equation A. Differential Equations to Model Chikungunya and Dengue Transmission Dynamics (adapted from Keeling and Rohani’s mosquito-borne disease SIR model. Equation B. Goodness of Fit Error (GOF) of Model Outputs For Target Data i at Time t over Time Period 1 to τ weeks. Table A. Distributions of Initial Parameter Sets for Calibration. Table B. Percent of Parameter Sets that Prefer Each Intervention, by Vaccine Cost (CV) and Diagnostic Test Cost (CD). Preferred strategy is defined as the intervention with the minimum incremental cost/DALY averted less than $18,132. If each incremental cost/DALY averted is greater than the WTP, the status quo is preferred; LLIN = long-lasting insecticide-treated nets; Routine Dengue Vaccination corresponds to vaccination of all 9-year-olds. * Base case. Table C. One-way Sensitivity Analysis of Insecticide and LLIN Efficacy, Cost, and Coverage. LLIN = long-lasting insecticide-treated nets. Table D. Scenario Analysis of Hypothetical Chikungunya Vaccine. NMB = Net monetary benefit, calculated assuming a willingness to pay of $18,132 per DALY; ICER = incremental cost per DALY averted, compared to status quo; Routine Dengue Vaccination corresponds to vaccination of all 9-year-olds. Table E. Scenario Analysis of Test and Vaccinate Strategy using the DENV Detect IgG ELISA Dengue Diagnostic Test and Dengvaxia. LLIN = long-lasting insecticide-treated nets; Routine Dengue Vaccination corresponds to vaccination of all 9-year-olds; NMB = Net monetary benefit, calculated assuming a willingness to pay of $18,132 per DALY; ICER = incremental cost per DALY averted, compared to status quo. Table F. Scenario Analysis of Test and Vaccinate Strategy with TAK-003 Dengue Vaccine. LLIN = long-lasting insecticide-treated nets; Routine Dengue Vaccination corresponds to vaccination of all 9-year-olds; NMB = Net monetary benefit, calculated assuming a willingness to pay of $18,132 per DALY; ICER = incremental cost per DALY averted, compared to status quo. Ta [file pntd.0010086.s001.docx]

**SUPPLEMENT**– **Prevention and Control of Chikungunya and Dengue in Colombia: A Cost-effectiveness Analysis**

Anneke L. Claypool, Margaret L. Brandeau, Jeremy D. Goldhaber-Fiebert

**Equations A.** Differential Equations to Model Chikungunya and Dengue Transmission Dynamics (adapted from Keeling and Rohani’s mosquito-borne disease SIR model[1])

- Susceptible Humans – Chikungunya, Susceptible Humans No Previous Infection – Dengue
  - - $\frac{dX_{H}^{C}X_{H0}^{D}}{dt}=v_{H}\Gamma_{H}-({rT}_{HM}^{C}\frac{Y_{M}^{C}}{\Gamma_{M}} {+rT_{HM}^{D}\frac{Y_{M}^{D}}{\Gamma_{M}}- {rT}_{HM}^{C}\frac{Y_{M}^{C}}{\Gamma_{M}}{rT}_{HM}^{D}\frac{Y_{M}^{D}}{\Gamma_{M}}) X}_{H}^{C}X_{H0}^{D}-$
    - $\frac{q_{D}\Gamma_{H}}{X_{H}^{D}}(1-\omega)X_{H}^{C}X_{H0}^{D}-\frac{q_{C}\Gamma_{H}}{X_{H}^{C}}X_{H}^{C}X_{H0}^{D}-\mu_{H}X_{H}^{C}X_{H0}^{D}$
- Infected, Asymptomatic Humans – Chikungunya, Susceptible Humans No Previous Infection – Dengue
  - - $\frac{dY_{H0}^{C}X_{H0}^{D}}{dt}=\left( 1-P_{sym}^{C} \right)rT_{HM}^{C}\frac{Y_{M}^{C}}{\Gamma_{M}}\left( 1-rT_{HM}^{D}\frac{Y_{M}^{D}}{\Gamma_{M}} \right)\left( X_{H}^{C}X_{H0}^{D}+\left( 1-\epsilon^{C} \right)V_{H}^{C}X_{H0}^{D} \right)-$
    - $({rT}_{HM}^{D}\frac{Y_{M}^{D}}{\Gamma_{M}}+\gamma^{C} - \gamma^{C}{rT}_{HM}^{D}\frac{Y_{M}^{D}}{\Gamma_{M}})Y_{H0}^{C}X_{H0}^{D}-\frac{q_{D}\Gamma_{H}}{X_{H}^{D}}(1-\omega)Y_{H0}^{C}X_{H0}^{D}-\mu_{H}Y_{H0}^{C}X_{H0}^{D}$
- Infected, Symptomatic Humans – Chikungunya, Susceptible Humans No Previous Infection – Dengue
  - - $\frac{dY_{H1}^{C}X_{H0}^{D}}{dt}=P_{sym}^{C}rT_{HM}^{C}\frac{Y_{M}^{C}}{\Gamma_{M}}\left( 1-rT_{HM}^{D}Y_{M}^{D} \right)\left( X_{H}^{C}X_{H0}^{D}+\left( 1-\epsilon^{C} \right)V_{H}^{C}X_{H0}^{D} \right)-$
    - $\left( rT_{HM}^{D}\frac{Y_{M}^{D}}{\Gamma_{M}}+ \gamma^{C} - \gamma^{C}{rT}_{HM}^{D}\frac{Y_{M}^{D}}{\Gamma_{M}} \right)Y_{H1}^{C}X_{H0}^{D}-\frac{q_{D}\Gamma_{H}}{X_{H}^{D}}(1-\omega)Y_{H1}^{C}X_{H0}^{D}-{h^{C}\mu}_{H}Y_{H1}^{C}X_{H0}^{D}$
- Recovered Humans – No Sequelae Chikungunya, Susceptible Humans No Previous Infection – Dengue
  - - $\frac{dR_{H0}^{C}X_{H0}^{D}}{dt}=\gamma^{C}\left( 1-rT_{HM}^{D}\frac{Y_{M}^{D}}{\Gamma_{M}} \right)\left( {Y_{H0}^{C}X_{H0}^{D}+\left( 1-\beta\right)Y}_{H1}^{C}X_{H0}^{D} \right)+z\left( 1-rT_{HM}^{D}\frac{Y_{M}^{D}}{\Gamma_{M}} \right)R_{H1}^{C}X_{H0}^{D}- rT_{HM}^{D}\frac{Y_{M}^{D}}{\Gamma_{M}}R_{H0}^{C}X_{H0}^{D}-\frac{q_{D}\Gamma_{H}}{X_{H}^{D}}(1-\omega)R_{H0}^{C}X_{H0}^{D}- \mu_{H}R_{H0}^{C}X_{H0}^{D}$
- Recovered Humans – Sequelae Chikungunya, Susceptible Humans No Previous Infection – Dengue
  - - $\frac{dR_{H1}^{C}X_{H0}^{D}}{dt}=\gamma^{C}\left( 1-rT_{HM}^{D}\frac{Y_{M}^{D}}{\Gamma_{M}} \right){\beta Y}_{H1}^{C}X_{H0}^{D}-(z+ rT_{HM}^{D}\frac{Y_{M}^{D}}{\Gamma_{M}}- zrT_{HM}^{D}\frac{Y_{M}^{D}}{\Gamma_{M}})R_{H1}^{C}X_{H0}^{D}-\frac{q_{D}\Gamma_{H}}{X_{H}^{D}}(1-\omega)R_{H1}^{C}X_{H0}^{D}- \mu_{H}R_{H1}^{C}X_{H0}^{D}$
- Susceptible Humans – Chikungunya, Susceptible Humans with Previous Infection – Dengue
  - - $\frac{dX_{H}^{C}X_{H1}^{D}}{dt}=\gamma^{D}(1-rT_{HM}^{C}\frac{Y_{M}^{C}}{\Gamma_{M}})(X_{H}^{C}Y_{H0}^{D}+X_{H}^{C}Y_{H1}^{D}) - (rT_{HM}^{C}\frac{Y_{M}^{C}}{\Gamma_{M}}+ lrT_{HM}^{D}\frac{Y_{M}^{D}}{\Gamma_{M}} - rT_{HM}^{C}\frac{Y_{M}^{C}}{\Gamma_{M}}lrT_{HM}^{D}\frac{Y_{M}^{D}}{\Gamma_{M}})X_{H}^{C}X_{H1}^{D}-\frac{q_{D}\Gamma_{H}}{X_{H}^{D}}\omega X_{H}^{C}X_{H1}^{D}-\frac{q_{C}\Gamma_{H}}{X_{H}^{C}}X_{H}^{C}X_{H1}^{D}-\mu_{H}X_{H}^{C}X_{H1}^{D}$
- Infected, Asymptomatic Humans – Chikungunya, Susceptible Humans with Previous Infection – Dengue
  - - $\frac{dY_{H0}^{C}X_{H1}^{D}}{dt}= \gamma^{D}\left( 1-\gamma^{C} \right)\left( Y_{H0}^{C}Y_{H0}^{D}+Y_{H0}^{C}Y_{H1}^{D} \right)+$
    - $\left( 1-P_{sym}^{C} \right)rT_{HM}^{C}\frac{Y_{M}^{C}}{\Gamma_{M}}\gamma^{D}\left( X_{H}^{C}Y_{H0}^{D}+ X_{H}^{C}Y_{H1}^{D}+ \left( 1-\epsilon^{C} \right){(V}_{H}^{C}Y_{H0}^{D}+ V_{H}^{C}Y_{H1}^{D} \right))+$
    - $\left( 1-P_{sym}^{C} \right)rT_{HM}^{C}\frac{Y_{M}^{C}}{\Gamma_{M}}\left( 1-lrT_{HM}^{D}\frac{Y_{M}^{D}}{\Gamma_{M}} \right)\left( X_{H}^{C}X_{H1}^{D}+ {\left( 1-\epsilon^{C} \right)V}_{H}^{C}X_{H1}^{D} \right)-$
    - $(lrT_{HM}^{D}\frac{Y_{M}^{D}}{\Gamma_{M}} +\gamma^{C}- lrT_{HM}^{D}\frac{Y_{M}^{D}}{\Gamma_{M}}\gamma^{C})Y_{H0}^{C}X_{H1}^{D}-\frac{q_{D}\Gamma_{H}}{X_{H}^{D}}\omega Y_{H0}^{C}X_{H1}^{D}-\mu_{H}Y_{H0}^{C}X_{H1}^{D}$
- Infected, Symptomatic Humans – Chikungunya, Susceptible Humans with Previous Infection – Dengue
  - - $\frac{dY_{H1}^{C}X_{H1}^{D}}{dt}=\gamma^{D}\left( 1-\gamma^{C} \right)\left( Y_{H1}^{C}Y_{H0}^{D} + Y_{H1}^{C}Y_{H1}^{D} \right)+$
    - $P_{sym}^{C} rT_{HM}^{C}\frac{Y_{M}^{C}}{\Gamma_{M}}\gamma^{D}(X_{H}^{C}Y_{H0}^{D}+ X_{H}^{C}Y_{H1}^{D}+ \left( 1-\epsilon^{C} \right){(V}_{H}^{C}Y_{H0}^{D}+ V_{H}^{C}Y_{H1}^{D})) + P_{sym}^{C} rT_{HM}^{C}\frac{Y_{M}^{C}}{\Gamma_{M}}(1-lrT_{HM}^{D}\frac{Y_{M}^{D}}{\Gamma_{M}}) (X_{H}^{C}X_{H1}^{D}+ \left( 1-\epsilon^{C} \right)V_{H}^{C}X_{H1}^{D}) -$
    - $(lrT_{HM}^{D}\frac{Y_{M}^{D}}{\Gamma_{M}} + \gamma^{C} -lrT_{HM}^{D}\frac{Y_{M}^{D}}{\Gamma_{M}}\gamma^{C})Y_{H1}^{C}X_{H1}^{D}-\frac{q_{D}\Gamma_{H}}{X_{H}^{D}}\omega Y_{H1}^{C}X_{H1}^{D}-{h^{C}\mu}_{H}Y_{H1}^{C}X_{H1}^{D}$
- Recovered Humans – No Sequelae Chikungunya, Susceptible Humans with Previous Infection – Dengue
  - - $\frac{dR_{H0}^{C}X_{H1}^{D}}{dt}=\gamma^{D}(R_{H0}^{C}Y_{H0}^{D} + R_{H0}^{C}Y_{H1}^{D}) + \gamma^{C}(1-lrT_{HM}^{D}\frac{Y_{M}^{D}}{\Gamma_{M}})(Y_{H0}^{C}X_{H1}^{D}+(1-\beta)Y_{H1}^{C}X_{H1}^{D})+ z(1-lrT_{HM}^{D}\frac{Y_{M}^{D}}{\Gamma_{M}})R_{H1}^{C}X_{H1}^{D} + z\gamma^{D}(R_{H1}^{C}Y_{H0}^{D}+R_{H1}^{C}Y_{H1}^{D}) + \gamma^{C}\gamma^{D}Y_{H0}^{C}Y_{H0}^{D}+{\gamma^{C}\gamma^{D}Y}_{H0}^{C}Y_{H1}^{D} + (1-\beta)\gamma^{C}\gamma^{D}(Y_{H1}^{C}Y_{H0}^{D}+Y_{H1}^{C}Y_{H1}^{D}) - lrT_{HM}^{D}\frac{Y_{M}^{D}}{\Gamma_{M}}R_{H0}^{C}X_{H1}^{D}-\frac{q_{D}\Gamma_{H}}{X_{H}^{D}}\omega R_{H0}^{C}X_{H1}^{D}- \mu_{H}R_{H0}^{C}X_{H1}^{D}$
- Recovered Humans – Sequelae Chikungunya, Susceptible Humans with Previous Infection – Dengue
  - - $\frac{dR_{H1}^{C}X_{H1}^{D}}{dt}=\gamma^{D}\left( 1-z \right)\left( R_{H1}^{C}Y_{H0}^{D}+R_{H1}^{C}Y_{H1}^{D} \right)+ \gamma^{C}\left( 1-lrT_{HM}^{D}\frac{Y_{M}^{D}}{\Gamma_{M}} \right)\beta Y_{H1}^{C}X_{H1}^{D} + \gamma^{C}\gamma^{D}\beta\left( Y_{H1}^{C}Y_{H0}^{D}+ Y_{H1}^{C}Y_{H1}^{D} \right)-\left( z+ lrT_{HM}^{D}\frac{Y_{M}^{D}}{\Gamma_{M}}- zlrT_{HM}^{D}\frac{Y_{M}^{D}}{\Gamma_{M}} \right)R_{H1}^{C}X_{H1}^{D}-$
    - $\frac{q_{D}\Gamma_{H}}{X_{H}^{D}}\omega R_{H1}^{C}X_{H1}^{D}- \mu_{H}R_{H1}^{C}X_{H1}^{D}$
- Susceptible Humans – Chikungunya, Infected Asymptomatic Humans – Dengue
  - - $\frac{dX_{H}^{C}Y_{H0}^{D}}{dt}=(1-rT_{HM}^{C}\frac{Y_{M}^{C}}{\Gamma_{M}})(rT_{HM}^{D}\frac{Y_{M}^{D}}{\Gamma_{M}}\left( 1-P_{sym}^{D0} \right)X_{H}^{C}X_{H0}^{D} +$
    - $\left( 1-rT_{HM}^{C}\frac{Y_{M}^{C}}{\Gamma_{M}} \right)\left( 1-P_{sym}^{D1} \right)lrT_{HM}^{D}\frac{Y_{M}^{D}}{\Gamma_{M}}X_{H}^{C}X_{H1}^{D} +$
    - $\left( 1-rT_{HM}^{C}\frac{Y_{M}^{C}}{\Gamma_{M}} \right)\left( 1-P_{sym}^{D1} \right)\left( 1-w \right)rT_{HM}^{D}\frac{Y_{M}^{D}}{\Gamma_{M}}\left( 1-\epsilon^{D} \right)X_{H}^{C}V_{H}^{D} -$
    - $(\gamma^{D} + rT_{HM}^{C}\frac{Y_{M}^{C}}{\Gamma_{M}} - \gamma^{D}rT_{HM}^{C}\frac{Y_{M}^{C}}{\Gamma_{M}})X_{H}^{C}Y_{H0}^{D}-\frac{q_{C}\Gamma_{H}}{X_{H}^{C}}X_{H}^{C}Y_{H0}^{D}-\mu_{H}X_{H}^{C}Y_{H0}^{D}$
- Infected, Asymptomatic Humans – Chikungunya, Infected Asymptomatic Humans – Dengue
  - - $\frac{dY_{H0}^{C}Y_{H0}^{D}}{dt}= \left( 1-P_{sym}^{C} \right)rT_{HM}^{C}\frac{Y_{M}^{C}}{\Gamma_{M}}\left( 1-\gamma^{D} \right) \left( X_{H}^{C}Y_{H0}^{D}+\left( 1-\epsilon^{C} \right)V_{H}^{C}Y_{H0}^{D} \right)+$
    - $\left( 1-\gamma^{C} \right)\left( 1-P_{sym}^{D0} \right)rT_{HM}^{D}\frac{Y_{M}^{D}}{\Gamma_{M}}Y_{H0}^{C}X_{H0}^{D} + \left( 1-\gamma^{C} \right)\left( 1-P_{sym}^{D1} \right)lrT_{HM}^{D}\frac{Y_{M}^{D}}{\Gamma_{M}} Y_{H0}^{C}X_{H1}^{D}+\left( 1-\gamma^{C} \right)\left( 1-P_{sym}^{D1} \right)\left( 1-w \right)rT_{HM}^{D}\frac{Y_{M}^{D}}{\Gamma_{M}}\left( 1-\epsilon^{D} \right)Y_{H0}^{C}V_{H}^{D}+$
    - $\left( 1-P_{sym}^{C} \right)rT_{HM}^{C}\frac{Y_{M}^{C}}{\Gamma_{M}}\left( 1-P_{sym}^{D0} \right)rT_{HM}^{D}\frac{Y_{M}^{D}}{\Gamma_{M}} {(X}_{H}^{C}X_{H0}^{D}+(1-\epsilon^{C})V_{H}^{C}X_{H0}^{D}) + \left( 1-P_{sym}^{C} \right)rT_{HM}^{C}\frac{Y_{M}^{C}}{\Gamma_{M}} \left( 1-P_{sym}^{D1} \right)lrT_{HM}^{D}\frac{Y_{M}^{D}}{\Gamma_{M}} (X_{H}^{C}X_{H1}^{D}+ (1-\epsilon^{C})V_{H}^{C}X_{H1}^{D})+ \left( 1-P_{sym}^{C} \right)rT_{HM}^{C}\frac{Y_{M}^{C}}{\Gamma_{M}} \left( 1-P_{sym}^{D1} \right)(1-w)lrT_{HM}^{D}\frac{Y_{M}^{D}}{\Gamma_{M}} ( (1-\epsilon^{D})X_{H}^{C}V_{H}^{D}+$
    - ${(1-\epsilon^{D})(1-\epsilon^{C})V}_{H}^{C}V_{H}^{D}) -\left( \gamma^{D}+ \gamma^{C}- \gamma^{D}\gamma^{C} \right) Y_{H0}^{C}Y_{H0}^{D}-\mu_{H}Y_{H0}^{C}Y_{H0}^{D}$
- Infected, Symptomatic Humans- Chikungunya, Infected Asymptomatic Humans – Dengue
  - - $\frac{dY_{H1}^{C}Y_{H0}^{D}}{dt}=P_{sym}^{C}rT_{HM}^{C}\frac{Y_{M}^{C}}{\Gamma_{M}}\left( 1-\gamma^{D} \right)\left( X_{H}^{C}Y_{H0}^{D}+\left( 1-\epsilon^{C} \right)V_{H}^{C}Y_{H0}^{D} \right)+$
    - $P_{sym}^{C}rT_{HM}^{C}\frac{Y_{M}^{C}}{\Gamma_{M}}\left( 1-P_{sym}^{D0} \right)rT_{HM}^{D}\frac{Y_{M}^{D}}{\Gamma_{M}}\left( X_{H}^{C}X_{H0}^{D} +\left( 1-\epsilon^{C} \right)V_{H}^{C}X_{H0}^{D} \right)+$
    - $P_{sym}^{C}rT_{HM}^{C}\frac{Y_{M}^{C}}{\Gamma_{M}}\left( 1-P_{sym}^{D1} \right)(1-w)lrT_{HM}^{D}\frac{Y_{M}^{D}}{\Gamma_{M}}\left( \left( 1-\epsilon^{D} \right)X_{H}^{C}V_{H}^{D}+ \left( 1-\epsilon^{C} \right)\left( 1-\epsilon^{D} \right)V_{H}^{C}V_{H}^{D} \right)+ P_{sym}^{C}rT_{HM}^{C}\frac{Y_{M}^{C}}{\Gamma_{M}} \left( 1-P_{sym}^{D1} \right)lrT_{HM}^{D}\frac{Y_{M}^{D}}{\Gamma_{M}}\left( X_{H}^{C}X_{H1}^{D}+ \left( 1-\epsilon^{C} \right)V_{H}^{C}X_{H1}^{D} \right)+\left( 1-\gamma^{C} \right)rT_{HM}^{D}\frac{Y_{M}^{D}}{\Gamma_{M}}\left( 1-P_{sym}^{D0} \right)Y_{H1}^{C}X_{H0}^{D}+\left( 1-\gamma^{C} \right)(1-w)lrT_{HM}^{D} \frac{Y_{M}^{C}}{\Gamma_{M}}\left( 1-P_{sym}^{D1} \right)\left( 1-\epsilon^{D} \right)Y_{H1}^{C}V_{H}^{D} + (1-\gamma^{C})lrT_{HM}^{D}\frac{Y_{M}^{D}}{\Gamma_{M}}(1-P_{sym}^{D1})Y_{H1}^{C}X_{H1}^{D} -(\gamma^{D}+ \gamma^{C}-\gamma^{D}\gamma^{C})Y_{H1}^{C}Y_{H0}^{D}-{h^{C}\mu}_{H}Y_{H1}^{C}Y_{H0}^{D}$
- Recovered Humans – No Sequelae Chikungunya, Infected Asymptomatic Humans – Dengue
  - - $\frac{dR_{H0}^{C}Y_{H0}^{D}}{dt}=\gamma^{C}\left( 1-\gamma^{D} \right)Y_{H0}^{C}Y_{H0}^{D} +\gamma^{C}\left( 1-\beta\right)\left( 1-\gamma^{D} \right)Y_{H1}^{C}Y_{H0}^{D}+$
    - $\gamma^{C}\left( 1-P_{sym}^{D0} \right)rT_{HM}^{D}\frac{Y_{M}^{D}}{\Gamma_{M}} Y_{H0}^{C}X_{H0}^{D} +\gamma^{C}\left( 1-P_{sym}^{D1} \right)\left( 1-w \right)lrT_{HM}^{D}\frac{Y_{M}^{D}}{\Gamma_{M}}\left( 1-\epsilon^{D} \right) Y_{H0}^{C}V_{H}^{D} +\gamma^{C}\left( 1-\beta\right)\left( 1-P_{sym}^{D1} \right)\left( 1-w \right)lrT_{HM}^{D}\frac{Y_{M}^{D}}{\Gamma_{M}}\left( 1-\epsilon^{D} \right) Y_{H1}^{C}V_{H}^{D}+$
    - $\gamma^{C}\left( 1-\beta\right)\left( 1-P_{sym}^{D0} \right)rT_{HM}^{D}\frac{Y_{M}^{D}}{\Gamma_{M}} Y_{H1}^{C}X_{H0}^{D}+ \gamma^{C}\left( 1-P_{sym}^{D1} \right)lrT_{HM}^{D}\frac{Y_{M}^{D}}{\Gamma_{M}} Y_{H0}^{C}X_{H1}^{D} +\gamma^{C}\left( 1-\beta\right)\left( 1-P_{sym}^{D1} \right)lrT_{HM}^{D}\frac{Y_{M}^{D}}{\Gamma_{M}} Y_{H1}^{C}X_{H1}^{D} +z \left( 1-\gamma^{D} \right)R_{H1}^{C}Y_{H0}^{D}+$
    - $\left( 1-P_{sym}^{D0} \right)rT_{HM}^{D}\frac{Y_{M}^{D}}{\Gamma_{M}}R_{H0}^{C}X_{H0}^{D}+\left( 1-P_{sym}^{D1} \right)lrT_{HM}^{D}\frac{Y_{M}^{D}}{\Gamma_{M}}R_{H0}^{C}X_{H1}^{D}+$
    - $\left( 1-P_{sym}^{D1} \right)(1-w)lrT_{HM}^{D}\frac{Y_{M}^{D}}{\Gamma_{M}}(1-\epsilon^{D})R_{H0}^{C}V_{H}^{D}+z\left( 1-P_{sym}^{D0} \right)rT_{HM}^{D}\frac{Y_{M}^{D}}{\Gamma_{M}} R_{H1}^{C}X_{H0}^{D}+z\left( 1-P_{sym}^{D1} \right)(1-w)lrT_{HM}^{D}\frac{Y_{M}^{D}}{\Gamma_{M}}\left( 1-\epsilon^{D} \right) R_{H1}^{C}V_{H}^{D}+ z\left( 1-P_{sym}^{D1} \right)lrT_{HM}^{D}\frac{Y_{M}^{D}}{\Gamma_{M}}R_{H1}^{C}X_{H1}^{D} -\gamma^{D}R_{H0}^{C}Y_{H0}^{D}- \mu_{H}R_{H0}^{C}Y_{H0}^{D}$
- Recovered Humans – Sequelae Chikungunya, Infected Asymptomatic Humans – Dengue
  - - $\frac{dR_{H1}^{C}Y_{H0}^{D}}{dt}=\gamma^{C}\beta\left( 1-\gamma^{D} \right)Y_{H1}^{C}Y_{H0}^{D} +\left( 1-z \right)\left( 1-P_{sym}^{D0} \right)rT_{HM}^{D}\frac{Y_{M}^{D}}{\Gamma_{M}}R_{H1}^{C}X_{H0}^{D} +\left( 1-z \right)\left( 1-P_{sym}^{D1} \right)\left( 1-w \right)lrT_{HM}^{D}\frac{Y_{M}^{D}}{\Gamma_{M}}\left( 1-\epsilon^{D} \right)R_{H1}^{C}V_{H}^{D}+$
    - $\left( 1-z \right)\left( 1-P_{sym}^{D1} \right)lrT_{HM}^{D}\frac{Y_{M}^{D}}{\Gamma_{M}}R_{H1}^{C}X_{H1}^{D} +\gamma^{C}\beta\left( 1-P_{sym}^{D0} \right)rT_{HM}^{D}\frac{Y_{M}^{D}}{\Gamma_{M}}Y_{H1}^{C}X_{H0}^{D} +\gamma^{C}\beta\left( 1-P_{sym}^{D1} \right)(1-w)rT_{HM}^{D}\frac{Y_{M}^{D}}{\Gamma_{M}}(1-\epsilon^{D})Y_{H1}^{C}V_{H}^{D}+\gamma^{C}\beta(1-P_{sym}^{D1})lrT_{HM}^{D}\frac{Y_{M}^{D}}{\Gamma_{M}}Y_{H1}^{C}X_{H1}^{D}-(\gamma^{D} +z-\gamma^{D}z)R_{H1}^{C}Y_{H0}^{D}- \mu_{H}R_{H1}^{C}Y_{H0}^{D}$
- Susceptible Humans – Chikungunya, Infected Symptomatic Humans – Dengue
  - - $\frac{dX_{H}^{C}Y_{H1}^{D}}{dt}=\left( 1-rT_{HM}^{C}\frac{Y_{M}^{C}}{\Gamma_{M}} \right)P_{sym}^{D0} rT_{HM}^{D}\frac{Y_{M}^{D}}{\Gamma_{M}}X_{H}^{C}X_{H0}^{D}+ \left( 1-rT_{HM}^{C}\frac{Y_{M}^{C}}{\Gamma_{M}} \right)P_{sym}^{D1}lrT_{HM}^{D}\frac{Y_{M}^{D}}{\Gamma_{M}}X_{H}^{C}X_{H1}^{D}+ \left( 1-rT_{HM}^{C}\frac{Y_{M}^{C}}{\Gamma_{M}} \right)P_{sym}^{D1}\left( 1-w \right)lrT_{HM}^{D}\frac{Y_{M}^{D}}{\Gamma_{M}}{\left( 1-\epsilon^{D} \right)X}_{H}^{C}V_{H}^{D}-$
    - $\left( \gamma^{D}+ rT_{HM}^{C}\frac{Y_{M}^{C}}{\Gamma_{M}}-\gamma^{D}rT_{HM}^{C}Y_{M}^{C} \right)X_{H}^{C}Y_{H1}^{D}-\frac{q_{C}\Gamma_{H}}{X_{H}^{C}}X_{H}^{C}Y_{H1}^{D}-{h^{D}\mu}_{H}X_{H}^{C}Y_{H1}^{D}$
- Infected, Asymptomatic Humans – Chikungunya, Infected Symptomatic Humans – Dengue
  - - $\frac{dY_{H0}^{C}Y_{H1}^{D}}{dt}= \left( 1-P_{sym}^{C} \right)rT_{HM}^{C}\frac{Y_{M}^{C}}{\Gamma_{M}}\left( 1-\gamma^{D} \right)X_{H}^{C}Y_{H1}^{D}+$
    - $\left( 1-P_{sym}^{C} \right)rT_{HM}^{C}\frac{Y_{M}^{C}}{\Gamma_{M}}\left( 1-\gamma^{D} \right)\left( 1-\epsilon^{C} \right)\frac{Y_{M}^{C}}{\Gamma_{M}}V_{H}^{C}Y_{H1}^{D}+$
    - $\left( 1-P_{sym}^{C} \right)rT_{HM}^{C}\frac{Y_{M}^{C}}{\Gamma_{M}}P_{sym}^{D0}rT_{HM}^{D}\frac{Y_{M}^{D}}{\Gamma_{M}}X_{H}^{C}X_{H0}^{D} +$
    - $\left( 1-P_{sym}^{C} \right)rT_{HM}^{C}\frac{Y_{M}^{C}}{\Gamma_{M}}P_{sym}^{D0}rT_{HM}^{D}\frac{Y_{M}^{D}}{\Gamma_{M}}{\left( 1-\epsilon^{C} \right)V}_{H}^{C}X_{H0}^{D} +$
    - $\left( 1-P_{sym}^{C} \right)rT_{HM}^{C}\frac{Y_{M}^{C}}{\Gamma_{M}}P_{sym}^{D1}\left( 1-w \right)lrT_{HM}^{D}\frac{Y_{M}^{D}}{\Gamma_{M}}\left( 1-\epsilon^{D} \right)X_{H}^{C}V_{H}^{D}+$
    - $\left( 1-P_{sym}^{C} \right)rT_{HM}^{C}\frac{Y_{M}^{C}}{\Gamma_{M}}P_{sym}^{D1}\left( 1-w \right)lrT_{HM}^{D}\frac{Y_{M}^{D}}{\Gamma_{M}}\left( 1-\epsilon^{C} \right)\left( 1-\epsilon^{D} \right)V_{H}^{C}V_{H}^{D}+$
    - $\left( 1-P_{sym}^{C} \right)rT_{HM}^{C}\frac{Y_{M}^{C}}{\Gamma_{M}}P_{sym}^{D1}lrT_{HM}^{D}\frac{Y_{M}^{D}}{\Gamma_{M}} X_{H}^{C}X_{H1}^{D}+$
    - $\left( 1-P_{sym}^{C} \right)rT_{HM}^{C}\frac{Y_{M}^{C}}{\Gamma_{M}}P_{sym}^{D1}lrT_{HM}^{D}\frac{Y_{M}^{D}}{\Gamma_{M}}\left( 1-\epsilon^{C} \right)V_{H}^{C}X_{H1}^{D} + \left( 1- \gamma^{C} \right)P_{sym}^{D0}rT_{HM}^{D}\frac{Y_{M}^{D}}{\Gamma_{M}}Y_{H0}^{C}X_{H0}^{D}+ \left( 1- \gamma^{C} \right)P_{sym}^{D1}(1-w)lrT_{HM}^{D}\frac{Y_{M}^{D}}{\Gamma_{M}}\left( 1-\epsilon^{D} \right)Y_{H0}^{C}V_{H}^{D} + \left( 1- \gamma^{C} \right)P_{sym}^{D1}lrT_{HM}^{D}\frac{Y_{M}^{D}}{\Gamma_{M}} Y_{H0}^{C}X_{H1}^{D} -(\gamma^{D}+ \gamma^{C}-\gamma^{D}\gamma^{C})Y_{H0}^{C}Y_{H1}^{D}-{h^{D}\mu}_{H}Y_{H1}^{C}Y_{H1}^{D}$
- Infected, Symptomatic Humans – Chikungunya, Infected Symptomatic Humans – Dengue
  - - $\frac{dY_{H1}^{C}Y_{H1}^{D}}{dt}=P_{sym}^{C}rT_{HM}^{C}\frac{Y_{M}^{C}}{\Gamma_{M}} \left( 1-\gamma^{D} \right)X_{H}^{C}Y_{H1}^{D} +P_{sym}^{C}rT_{HM}^{C}\frac{Y_{M}^{C}}{\Gamma_{M}} \left( 1-\gamma^{D} \right)\left( 1-\epsilon^{C} \right)V_{H}^{C}Y_{H1}^{D} + P_{sym}^{C}rT_{HM}^{C}\frac{Y_{M}^{C}}{\Gamma_{M}}P_{sym}^{D0}rT_{HM}^{D}\frac{Y_{M}^{D}}{\Gamma_{M}} X_{H}^{C}X_{H0}^{D} + P_{sym}^{C}rT_{HM}^{C}\frac{Y_{M}^{C}}{\Gamma_{M}}P_{sym}^{D0}rT_{HM}^{D}\frac{Y_{M}^{D}}{\Gamma_{M}}\left( 1-\epsilon^{C} \right)V_{H}^{C}X_{H0}^{D} + P_{sym}^{C}rT_{HM}^{C}\frac{Y_{M}^{C}}{\Gamma_{M}}P_{sym}^{D1}\left( 1-w \right)lrT_{HM}^{D}\frac{Y_{M}^{D}}{\Gamma_{M}}\left( 1-\epsilon^{D} \right)X_{H}^{C}V_{H}^{D}+$
    - $P_{sym}^{C}rT_{HM}^{C}\frac{Y_{M}^{C}}{\Gamma_{M}}P_{sym}^{D1}(1-w)lrT_{HM}^{D}\frac{Y_{M}^{D}}{\Gamma_{M}}\left( 1-\epsilon^{C} \right)\left( 1-\epsilon^{D} \right)V_{H}^{C}V_{H}^{D}+ P_{sym}^{C}rT_{HM}^{C}\frac{Y_{M}^{C}}{\Gamma_{M}}P_{sym}^{D1} lrT_{HM}^{D}\frac{Y_{M}^{D}}{\Gamma_{M}}X_{H}^{C}X_{H1}^{D} + P_{sym}^{C}rT_{HM}^{C}\frac{Y_{M}^{C}}{\Gamma_{M}}P_{sym}^{D1} lrT_{HM}^{D}\frac{Y_{M}^{D}}{\Gamma_{M}}{\left( 1-\epsilon^{C} \right)V}_{H}^{C}X_{H1}^{D}+ \left( 1-\gamma^{C} \right)P_{sym}^{D0}rT_{HM}^{D}\frac{Y_{M}^{D}}{\Gamma_{M}}Y_{H1}^{C}X_{H0}^{D}+ \left( 1-\gamma^{C} \right)P_{sym}^{D1}(1-w)lrT_{HM}^{D}\frac{Y_{M}^{D}}{\Gamma_{M}}{\left( 1-\epsilon^{D} \right)Y}_{H1}^{C}V_{H}^{D} + \left( 1-\gamma^{C} \right)P_{sym}^{D1}lrT_{HM}^{D}\frac{Y_{M}^{D}}{\Gamma_{M}} Y_{H1}^{C}X_{H1}^{D} -(\gamma^{D}+ \gamma^{C}-\gamma^{D}\gamma^{C})Y_{H1}^{C}Y_{H1}^{D}-h^{D}{h^{C}\mu}_{H}Y_{H1}^{C}Y_{H1}^{D}$
- Recovered Humans – No Sequelae Chikungunya, Infected Symptomatic Humans – Dengue
  - - $\frac{dR_{H0}^{C}Y_{H1}^{D}}{dt}=\gamma^{C}\left( 1-\gamma^{D} \right)Y_{H0}^{C}Y_{H1}^{D} +\gamma^{C}\left( 1-\beta\right)\left( 1-\gamma^{D} \right)Y_{H1}^{C}Y_{H1}^{D}+ P_{sym}^{D0} rT_{HM}^{D}\frac{Y_{M}^{D}}{\Gamma_{M}}R_{H0}^{C}X_{H0}^{D}+ P_{sym}^{D1} \left( 1-w \right)lrT_{HM}^{D}\frac{Y_{M}^{D}}{\Gamma_{M}}\left( 1-\epsilon^{D} \right)R_{H0}^{C}V_{H}^{D} + P_{sym}^{D1}lrT_{HM}^{D}\frac{Y_{M}^{D}}{\Gamma_{M}} R_{H0}^{C}X_{H1}^{D}+$
    - $z\left( 1-\gamma^{D} \right)R_{H1}^{C}Y_{H1}^{D} + zP_{sym}^{D0}rT_{HM}^{D}\frac{Y_{M}^{D}}{\Gamma_{M}} R_{H1}^{C}X_{H0}^{D} +$
    - $zP_{sym}^{D1}\left( 1-w \right)lrT_{HM}^{D}\frac{Y_{M}^{D}}{\Gamma_{M}}\left( 1-\epsilon^{D} \right)R_{H1}^{C}V_{H}^{D} + zP_{sym}^{D1}lrT_{HM}^{D}\frac{Y_{M}^{D}}{\Gamma_{M}} R_{H1}^{C}X_{H1}^{D} + \gamma^{C}P_{sym}^{D0}rT_{HM}^{D}\frac{Y_{M}^{D}}{\Gamma_{M}} Y_{H0}^{C}X_{H0}^{D}+\gamma^{C}\left( 1-\beta\right)P_{sym}^{D0}rT_{HM}^{D}\frac{Y_{M}^{D}}{\Gamma_{M}} Y_{H1}^{C}X_{H0}^{D} +$
    - $\gamma^{C}P_{sym}^{D1}\left( 1-w \right)lrT_{HM}^{D}\frac{Y_{M}^{D}}{\Gamma_{M}}{\left( 1-\epsilon^{D} \right)Y}_{H0}^{C}V_{H}^{D}+$
    - $\gamma^{C}\left( 1-\beta\right)P_{sym}^{D1}\left( 1-w \right)lrT_{HM}^{D}\frac{Y_{M}^{D}}{\Gamma_{M}}\left( 1-\epsilon^{D} \right)Y_{H1}^{C}V_{H}^{D} + \gamma^{C}lrT_{HM}^{D}\frac{Y_{M}^{D}}{\Gamma_{M}}P_{sym}^{D1}Y_{H0}^{C}X_{H1}^{D}+$
    - $\gamma^{C}(1-\beta)lrT_{HM}^{D}\frac{Y_{M}^{D}}{\Gamma_{M}}P_{sym}^{D1} Y_{H1}^{C}X_{H1}^{D} -\gamma^{D}R_{H0}^{C}Y_{H1}^{D}{-h^{D}\mu}_{H}R_{H0}^{C}Y_{H1}^{D}$
- Recovered Humans – Sequelae Chikungunya, Infected Symptomatic Humans – Dengue
  - - $\frac{dR_{H1}^{C}Y_{H1}^{D}}{dt}=\gamma^{C}\beta\left( 1-\gamma^{D} \right)Y_{H1}^{C}Y_{H1}^{D}+ \left( 1-z \right)P_{sym}^{D0}rT_{HM}^{D}\frac{Y_{M}^{D}}{\Gamma_{M}}R_{H1}^{C}X_{H0}^{D} +$
    - $\left( 1-z \right)P_{sym}^{D1}(1-w)lrT_{HM}^{D}\frac{Y_{M}^{D}}{\Gamma_{M}}{\left( 1-\epsilon^{D} \right)R}_{H1}^{C}V_{H}^{D} + \left( 1-z \right)P_{sym}^{D1}lrT_{HM}^{D}\frac{Y_{M}^{D}}{\Gamma_{M}}R_{H1}^{C}X_{H1}^{D} + \gamma^{C}\beta P_{sym}^{D0}rT_{HM}^{D}\frac{Y_{M}^{D}}{\Gamma_{M}} Y_{H1}^{C}X_{H0}^{D} + \gamma^{C}\beta P_{sym}^{D1}(1-w)lrT_{HM}^{D}\frac{Y_{M}^{D}}{\Gamma_{M}} \left( 1-\epsilon^{D} \right)Y_{H1}^{C}V_{H}^{D}+ \gamma^{C}\beta P_{sym}^{D1}lrT_{HM}^{D}\frac{Y_{M}^{D}}{\Gamma_{M}}Y_{H1}^{C}X_{H1}^{D} -(\gamma^{D}+ z -\gamma_{H}^{D}z )R_{H1}^{C}Y_{H1}^{D}- h^{D}\mu_{H}R_{H1}^{C}Y_{H1}^{D}$
- Vaccine Humans – Chikungunya, Susceptible Humans No Previous Infection – Dengue
  - - $\frac{dV_{H}^{C}X_{H0}^{D}}{dt}=\frac{q_{C}\Gamma_{H}}{X_{H}^{C}}X_{H}^{C}X_{H0}^{D}-$
    - $(\left( 1-\epsilon^{C} \right)rT_{HM}^{C}\frac{Y_{M}^{C}}{\Gamma_{M}}{+rT_{HM}^{D}\frac{Y_{M}^{D}}{\Gamma_{M}}-\left( 1-\epsilon^{C} \right){rT}_{HM}^{C}\frac{Y_{M}^{C}}{\Gamma_{M}}{rT}_{HM}^{D}\frac{Y_{M}^{D}}{\Gamma_{M}}) V}_{H}^{C}X_{H0}^{D}-$
    - $\frac{q_{D}\Gamma_{H}}{X_{H}^{D}}\left( 1-\omega\right)V_{H}^{C}X_{H0}^{D}-\mu_{H}V_{H}^{C}X_{H0}^{D}$
- Vaccine Humans – Chikungunya, Susceptible Humans with Previous Infection – Dengue
  - - $\frac{dV_{H}^{C}X_{H1}^{D}}{dt}= \frac{q_{C}\Gamma_{H}}{X_{H}^{C}}X_{H}^{C}X_{H1}^{D}+\left( 1-\left( \left( 1-\epsilon^{C} \right)rT_{HM}^{C}\frac{Y_{M}^{C}}{\Gamma_{M}} \right) \right){\gamma^{D}V}_{H}^{C}Y_{H0}^{D}+$
    - $\left( 1-\left( \left( 1-\epsilon^{C} \right)rT_{HM}^{C}\frac{Y_{M}^{C}}{\Gamma_{M}} \right) \right){\gamma^{D}V}_{H}^{C}Y_{H1}^{D}-$
    - $\left( \left( 1-\epsilon^{C} \right)rT_{HM}^{C}\frac{Y_{M}^{C}}{\Gamma_{M}}+ lrT_{HM}^{D}\frac{Y_{M}^{D}}{\Gamma_{M}} - \left( 1-\epsilon^{C} \right)rT_{HM}^{C}\frac{Y_{M}^{C}}{\Gamma_{M}}lrT_{HM}^{D}\frac{Y_{M}^{D}}{\Gamma_{M}} \right)V_{H}^{C}X_{H1}^{D}- \frac{q_{D}\Gamma_{H}}{X_{H}^{D}}\psi V_{H}^{C}X_{H1}^{D}-\mu_{H}V_{H}^{C}X_{H1}^{D}$
- Vaccine Humans – Chikungunya, Infected Asymptomatic Humans – Dengue
  - - $\frac{dV_{H}^{C}Y_{H0}^{D}}{dt}= \frac{q_{C}\Gamma_{H}}{X_{H}^{C}}X_{H}^{C}Y_{H0}^{D}+$
    - $\left( 1-\left( \left( 1-\epsilon^{C} \right)rT_{HM}^{C}\frac{Y_{M}^{C}}{\Gamma_{M}} \right) \right){{(1-P}_{sym}^{D1})\left( 1-\epsilon^{D} \right)(1-w)lrT_{HM}^{D}\frac{Y_{M}^{D}}{\Gamma_{M}}V}_{H}^{C}V_{H}^{D}+\left( 1-\left( \left( 1-\epsilon^{C} \right)rT_{HM}^{C}\frac{Y_{M}^{C}}{\Gamma_{M}} \right) \right){{(1-P}_{sym}^{D0})rT_{HM}^{D}\frac{Y_{M}^{D}}{\Gamma_{M}}V}_{H}^{C}X_{H0}^{D}+$
    - $\left( 1-\left( \left( 1-\epsilon^{C} \right)rT_{HM}^{C}\frac{Y_{M}^{C}}{\Gamma_{M}} \right) \right){{(1-P}_{sym}^{D1})lrT_{HM}^{D}\frac{Y_{M}^{D}}{\Gamma_{M}}V}_{H}^{C}X_{H1}^{D}-(\gamma^{D} + (1-\epsilon^{C})rT_{HM}^{C}\frac{Y_{M}^{C}}{\Gamma_{M}} - \gamma^{D}(1-\epsilon^{C})rT_{HM}^{C}\frac{Y_{M}^{C}}{\Gamma_{M}})V_{H}^{C}Y_{H0}^{D}-\mu_{H}V_{H}^{C}Y_{H0}^{D}$
- Vaccine Humans – Chikungunya, Infected Symptomatic Humans – Dengue
  - - $\frac{dV_{H}^{C}Y_{H1}^{D}}{dt}=\frac{q_{C}\Gamma_{H}}{X_{H}^{C}}X_{H}^{C}Y_{H1}^{D}+$
    - $\left( 1-\left( \left( 1-\epsilon^{C} \right)rT_{HM}^{C}\frac{Y_{M}^{C}}{\Gamma_{M}} \right) \right){P_{sym}^{D1}\left( 1-\epsilon^{D} \right)\left( 1-w \right)lrT_{HM}^{D}\frac{Y_{M}^{D}}{\Gamma_{M}}V}_{H}^{C}V_{H}^{D}+$
    - $\left( 1-\left( \left( 1-\epsilon^{C} \right)rT_{HM}^{C}\frac{Y_{M}^{C}}{\Gamma_{M}} \right) \right){P_{sym}^{D0}rT_{HM}^{D}\frac{Y_{M}^{D}}{\Gamma_{M}}V}_{H}^{C}X_{H0}^{D}+$
    - $\left( 1-\left( \left( 1-\epsilon^{C} \right)rT_{HM}^{C}\frac{Y_{M}^{C}}{\Gamma_{M}} \right) \right){P_{sym}^{D1}lrT_{HM}^{D}\frac{Y_{M}^{D}}{\Gamma_{M}}V}_{H}^{C}X_{H1}^{D}-$
    - $\left( \gamma^{D}+ (1-\epsilon^{C})rT_{HM}^{C}\frac{Y_{M}^{C}}{\Gamma_{M}}-\gamma^{D}(1-\epsilon^{C})rT_{HM}^{C}\frac{Y_{M}^{C}}{\Gamma_{M}} \right)V_{H}^{C}Y_{H1}^{D}-{h^{D}\mu}_{H}V_{H}^{C}Y_{H1}^{D}$
- Susceptible Humans – Chikungunya, Vaccine Humans – Dengue
  - - $\frac{dX_{H}^{C}V_{H}^{D}}{dt}= \frac{q_{D}\Gamma_{H}}{X_{H}^{D}}\left( 1-\omega\right)X_{H}^{C}X_{H0}^{D}+ \frac{q_{D}\Gamma_{H}}{X_{H}^{D}}\psi X_{H}^{C}X_{H1}^{D}-$
    - $(rT_{HM}^{C}\frac{Y_{M}^{C}}{\Gamma_{M}}{+(1-\epsilon^{D})(1-w){rT}_{HM}^{D}\frac{Y_{M}^{D}}{\Gamma_{M}}- {rT}_{HM}^{C}\frac{Y_{M}^{C}}{\Gamma_{M}}{(1-\epsilon^{D})(1-w)rT}_{HM}^{D}\frac{Y_{M}^{D}}{\Gamma_{M}}) X}_{H}^{C}V_{H}^{D}-\frac{q_{C}\Gamma_{H}}{X_{H}^{C}}X_{H}^{C}V_{H}^{D}-\mu_{H}X_{H}^{C}V_{H}^{D}$
- Infected, Asymptomatic Humans – Chikungunya, Vaccine Humans – Dengue
  - - $\frac{dY_{H0}^{C}V_{H}^{D}}{dt}= \frac{q_{D}\Gamma_{H}}{X_{H}^{D}}\left( 1-\omega\right)Y_{H0}^{C}X_{H0}^{D}+ \frac{q_{D}\Gamma_{H}}{X_{H}^{D}}\psi Y_{H0}^{C}X_{H1}^{D}+$
    - $\left( 1-\epsilon^{C} \right)rT_{HM}^{C}\frac{Y_{M}^{C}}{\Gamma_{M}}\left( 1-P_{Sym}^{C} \right)\left( 1-\left( \left( 1-\epsilon^{D} \right)l{rT}_{HM}^{D}\frac{Y_{M}^{D}}{\Gamma_{M}} \right) \right)V_{H}^{C}V_{H}^{D}+$
    - $rT_{HM}^{C}\frac{Y_{M}^{C}}{\Gamma_{M}}(1-P_{Sym}^{C})(1-(\left( 1-\epsilon^{D} \right){rT}_{HM}^{D}\frac{Y_{M}^{D}}{\Gamma_{M}}))X_{H}^{C}V_{H}^{D}-((1-\epsilon^{D})(1-w){rT}_{HM}^{D}\frac{Y_{M}^{D}}{\Gamma_{M}}+\gamma^{C} - \gamma^{C}{(1-\epsilon^{D})(1-w)rT}_{HM}^{D}\frac{Y_{M}^{D}}{\Gamma_{M}})Y_{H0}^{C}V_{H}^{D}-\mu_{H}Y_{H0}^{C}V_{H}^{D}$
- Infected, Symptomatic Humans – Chikungunya, Vaccine Humans – Dengue
  - - $\frac{dY_{H1}^{C}V_{H}^{D}}{dt}= \frac{q_{D}\Gamma_{H}}{X_{H}^{D}}\left( 1-\omega\right)Y_{H1}^{C}X_{H0}^{D}+ \frac{q_{D}\Gamma_{H}}{X_{H}^{D}}\psi Y_{H1}^{C}X_{H1}^{D}+$
    - $\left( 1-\epsilon^{C} \right)rT_{HM}^{C}\frac{Y_{M}^{C}}{\Gamma_{M}}P_{Sym}^{C}\left( 1-\left( \left( 1-\epsilon^{D} \right){lrT}_{HM}^{D}\frac{Y_{M}^{D}}{\Gamma_{M}} \right) \right)V_{H}^{C}V_{H}^{D}+$
    - $rT_{HM}^{C}\frac{Y_{M}^{C}}{\Gamma_{M}}P_{Sym}^{C}\left( 1-\left( \left( 1-\epsilon^{D} \right){lrT}_{HM}^{D}\frac{Y_{M}^{D}}{\Gamma_{M}} \right) \right)X_{H}^{C}V_{H}^{D}-$
    - $\left( \left( 1-\epsilon^{D} \right)lr\left( 1-w \right)T_{HM}^{D}\frac{Y_{M}^{D}}{\Gamma_{M}}+ \gamma^{C} - \gamma^{C}{(1-\epsilon^{D})(1-w)lrT}_{HM}^{D}\frac{Y_{M}^{D}}{\Gamma_{M}} \right)Y_{H1}^{C}V_{H}^{D}-{h^{C}\mu}_{H}Y_{H1}^{C}V_{H}^{D}$
- Recovered Humans – No Sequelae Chikungunya, Vaccine Humans – Dengue
  - - $\frac{dR_{H0}^{C}V_{H}^{D}}{dt}=\frac{q_{D}\Gamma_{H}}{X_{H}^{D}}\left( 1-\omega\right)R_{H0}^{C}X_{H0}^{D}+ \frac{q_{D}\Gamma_{H}}{X_{H}^{D}}\psi R_{H0}^{C}X_{H1}^{D}+$
    - $\gamma^{C}\left( 1-\left( \left( 1-\epsilon^{D} \right)l{rT}_{HM}^{D}\frac{Y_{M}^{D}}{\Gamma_{M}} \right) \right)Y_{H0}^{C}V_{H}^{D}+ \gamma^{C}(1-\beta)(1-(\left( 1-\epsilon^{D} \right){rT}_{HM}^{D}\frac{Y_{M}^{D}}{\Gamma_{M}}))Y_{H1}^{C}V_{H}^{D}+ z(1-(\left( 1-\epsilon^{D} \right){lrT}_{HM}^{D}\frac{Y_{M}^{D}}{\Gamma_{M}}))R_{H1}^{C}V_{H}^{D}- (1-\epsilon^{D})(1-w)lrT_{HM}^{D}\frac{Y_{M}^{D}}{\Gamma_{M}}R_{H0}^{C}V_{H}^{D}- \mu_{H}R_{H0}^{C}V_{H}^{D}$
- Recovered Humans – Sequelae Chikungunya, Vaccine Humans – Dengue
  - - $\frac{dR_{H1}^{C}V_{H}^{D}}{dt}= \frac{q_{D}\Gamma_{H}}{X_{H}^{D}}\left( 1-\omega\right)R_{H1}^{C}X_{H0}^{D}+ \frac{q_{D}\Gamma_{H}}{X_{H}^{D}}\psi R_{H1}^{C}X_{H1}^{D}+$
    - $\gamma^{C}\beta(1-(\left( 1-\epsilon^{D} \right){lrT}_{HM}^{D}\frac{Y_{M}^{D}}{\Gamma_{M}}))Y_{H1}^{C}V_{H}^{D}-(z+ (1-\epsilon^{D})lrT_{HM}^{D}\frac{Y_{M}^{D}}{\Gamma_{M}}-$
    - $z(1-\epsilon^{D})lrT_{HM}^{D}\frac{Y_{M}^{D}}{\Gamma_{M}})(1-w)R_{H1}^{C}V_{H}^{D}- \mu_{H}R_{H1}^{C}V_{H}^{D}$
- Vaccine Humans – Chikungunya, Vaccine Humans – Dengue
  - - $\frac{dV_{H}^{C}V_{H}^{D}}{dt}=\frac{q_{C}\Gamma_{H}}{X_{H}^{C}}X_{H}^{C}V_{H}^{D}+\frac{q_{D}\Gamma_{H}}{X_{H}^{D}}\left( 1-\omega\right)V_{H}^{C}X_{H0}^{D}+ \frac{q_{D}\Gamma_{H}}{X_{H}^{D}}\psi V_{H}^{C}X_{H1}^{D}-$
    - $((1-\epsilon^{C})rT_{HM}^{C}\frac{Y_{M}^{C}}{\Gamma_{M}}{+ {\left( 1-\epsilon^{D} \right)\left( 1-w \right)lrT}_{HM}^{D}\frac{Y_{M}^{D}}{\Gamma_{M}}- (1-\epsilon^{C}){rT}_{HM}^{C}\frac{Y_{M}^{C}}{\Gamma_{M}}{(1-\epsilon^{D})(1-w)lrT}_{HM}^{D}\frac{Y_{M}^{D}}{\Gamma_{M}}) V}_{H}^{C}V_{H}^{D}-\mu_{H}V_{H}^{C}V_{H}^{D}$
- Susceptible Mosquitos – Chikungunya, Susceptible Mosquitos – Dengue
  - - $\frac{dX_{M}^{C}X_{M}^{D}}{dt}=v_{M}\Gamma_{M}-(rT_{MH}^{C}\frac{\left( Y_{H0}^{C}+Y_{H1}^{C} \right)}{\Gamma_{H}}+rT_{MH}^{D}\frac{\left( Y_{H0}^{D}+Y_{H1}^{D} \right)}{\Gamma_{H}}-r^{2}T_{MH}^{C}\frac{\left( Y_{H0}^{C}+Y_{H1}^{C} \right)}{\Gamma_{H}} T_{MH}^{D}\frac{\left( Y_{H0}^{D}+Y_{H1}^{D} \right)}{\Gamma_{H}})X_{M}^{C}X_{M}^{D}-\mu_{M}(1+\eta)X_{M}^{C}X_{M}^{D}$
- Infected Mosquitos – Chikungunya, Susceptible Mosquitos – Dengue
  - - $\frac{dY_{M}^{C}X_{M}^{D}}{dt}=rT_{MH}^{C}\frac{Y_{H0}^{C}+Y_{H1}^{C}}{\Gamma_{H}}\left( 1-rT_{MH}^{D}\frac{\left( Y_{H0}^{D}+Y_{H1}^{D} \right)}{\Gamma_{H}} \right)X_{M}^{C}X_{M}^{D}-rT_{MH}^{D}\frac{\left( Y_{H0}^{D}+Y_{H1}^{D} \right)}{\Gamma_{H}} Y_{M}^{C}X_{M}^{D}-$
    - $\mu_{M}(1+\eta)Y_{M}^{C}X_{M}^{D}$
- Susceptible Mosquitos – Chikungunya, Infected Mosquitos – Dengue
  - - $\frac{dX_{M}^{C}Y_{M}^{D}}{dt}=\left( 1-rT_{MH}^{C} \frac{\left( Y_{H0}^{C}+Y_{H1}^{C} \right)}{\Gamma_{H}} \right)rT_{MH}^{D}\frac{\left( Y_{H0}^{D}+Y_{H1}^{D} \right)}{\Gamma_{H}}X_{M}^{C}X_{M}^{D}-rT_{MH}^{C}\frac{\left( Y_{H0}^{C}+Y_{H1}^{C} \right)}{\Gamma_{H}} X_{M}^{C}Y_{M}^{D}-$
    - $\mu_{M}(1+\eta)X_{M}^{C}Y_{M}^{D}$
- Infected Mosquitos – Chikungunya, Infected Mosquitos – Dengue
  - - $\frac{dY_{M}^{C}Y_{M}^{D}}{dt}=rT_{MH}^{C}\frac{\left( Y_{H0}^{C}+Y_{H1}^{C} \right)}{\Gamma_{H}}X_{M}^{C}Y_{M}^{D}+rT_{MH}^{D}\frac{\left( Y_{H0}^{D}+Y_{H1}^{D} \right)}{\Gamma_{H}}Y_{M}^{C}X_{M}^{D}+rT_{MH}^{C}\frac{\left( Y_{H0}^{C}+Y_{H1}^{C} \right)}{\Gamma_{H}}rT_{MH}^{D}\frac{\left( Y_{H0}^{D}+Y_{H1}^{D} \right)}{\Gamma_{H}}X_{M}^{C}X_{M}^{D}-\mu_{M}(1+\eta)Y_{M}^{C}Y_{M}^{D}$

Variables

$v_{i}$ = birth rate of species *i*

*r* = biting rate

$T_{ij}$ = transmission probability to species *i* from *j* for disease *k*

$\mu_{i}$ = per capita death rate of species *i*

$\gamma^{k}$ = recovery rate for humans for disease *k*

*l*  = limit on reinfection of dengue

$P_{sym}^{C}$ = probability of chikungunya symptoms

$P_{sym}^{D0}$ = probability of dengue symptoms with no previous infection

$P_{sym}^{D1}$ = probability of dengue symptoms with previous infection

*h^k^* = hazard ratio for disease *k*

$\beta$ = probability of sequelae given symptoms, chikungunya

*z* = probability of sequelae recovery, chikungunya

$\Gamma_{i}$ = total population of species *i*

$\epsilon^{k}$ = efficacy of vaccine for disease *k*

$\eta^{k}$ = efficacy of insecticide for disease *k*

*q_i_* = population vaccination rate for disease *i*

ψ = sensitivity of dengue seropositive test

ω = specificity of dengue seropositive test

*w* = probability of continued vaccine protection after infection

$X_{i\lambda}^{k}$ = population of susceptible of species *i* for disease k with previous infection $\lambda$

($\lambda=0$ is no previous infection, $\lambda=1$ is with previous infection)*

$Y_{i\theta}^{k}$ = population of infected of species *i* for disease k with symptoms $\theta$

($\theta=0$ is no symptoms, $\theta=1$ is with symptoms)*

$R_{i\rho}^{C}$ = population of recovered of species *i* for chikungunya with sequelae $\rho$

($\rho=0$ is no sequelae, $\rho=1$ is with sequelae)*

$V_{H}^{k}$ = population of vaccinated humans for disease *k**

_________________________

*In the combined model, states are designated by chikungunya status and dengue status. For example, $X_{H}^{C}X_{H0}^{D}$ refers to the population of people who are both susceptible for chikungunya and susceptible with no previous infection for dengue. $X_{H}^{D}$ refers to the sum of all susceptible dengue states.

**Equation B**. Goodness of Fit Error (GOF) of Model Outputs For Target Data *i* at Time *t* over Time Period 1 to *τ* weeks.[2]

$$GOF\left( x \right)= 4 \sum_{1}^{\tau} |O_{c}^{x}\left( t \right)-M_{c}^{x}\left( t \right)|+ \sum_{1}^{\tau} \left| O_{d}^{x}\left( t \right)-M_{d}^{x}\left( t \right) \right| + \frac{max\left( O_{c}^{x}\left( t \right) \right)}{8} [|max(O_{i}^{x})-max(M_{i}^{x})|+ \left| T\left( O_{i}^{x} \right)-T\left( M_{i}^{x} \right) \right|]$$

$$GOF = GOF\left( chikungunya \right)+GOF\left( dengue \right)$$

Variables

*x* = disease

*τ* = maximum time of observation period, in weeks

*t* = time, in weeks

*c* = cumulative incidence

*d* = death

*i* = absolute incidence

*T* = time *t* when incidence is equal to the maximum incidence

$O_{y}^{x}\left( t \right)$ = observed value for disease *x* outcome *y* at time *t*

$M_{y}^{x}\left( t \right)$ = model output for disease *x* outcome *y* at time *t*

We weighted cumulative incidence four times greater than cumulative deaths, as incidence of disease contributes directly to disease-related deaths. Maximum incidence and time of maximum incidence is weighted with the highest cumulative incidence, divided by 8 to account for the two points of peak weekly incidence.

**Table A. Distributions of Initial Parameter Sets for Calibration**

| **Variable** | **Estimated Value** | **Prior Range** | **Prior Distribution(3, 4)** | **Source** |
| --- | --- | --- | --- | --- |
| Mosquito death rate/week |  | (0,1] | Beta(0.47,2.16) |  |
| Mosquito birth rate/week |  | (0,1] | Beta(0.47,2.16) |  |
| Initial mosquito population |  | (0, 48321000*4] | Uniform(1000, 193284000) |  |
| Biting rate/day | 0.33-1/day | (0, 11] | Lognormal(1.50, 0.23) | [5, 6] |
| Probability of chikungunya symptoms | 0.723 - 0.833 | (0,1] | Lognormal(0.36,0.14) | [6] |
| Probability of chikungunya transmission from human to mosquito after bite |  | [0.1,0.9] | Uniform(0.1,0.9) |  |
| Probability of chikungunya transmission from mosquito to human after bite | 0.06–0.6 | [0.1,0.9] | Uniform(0.1,0.9) | [5, 6] |
| Probability of chikungunya recovery in humans |  | [0.3,0.7] | Uniform(0.3,0.7) |  |
| Hazard ratio of death during chikungunya infection |  | [1,10] | Uniform(1,10) |  |
| Probability of dengue transmission from human to mosquito after bite | 0.5-1 | [0.1,0.9] | Uniform(0.1,0.9) | [7] |
| Probability of dengue transmission from mosquito to human after bite | 0.1-0.75 | [0.1,0.9] | Uniform(0.1,0.9) | [7] |
| Probability of dengue recovery in humans |  | [0.3,0.7] | Uniform(0.3,0.7) |  |
| Probability of dengue symptoms given no previous infection | 0.18 | (0,1] | Lognormal(0.69,0.24) | [8] |
| Probability of dengue symptoms given previous infection | 0.41 | (0,1] | Lognormal (-0.69,0.24) | [8] |
| Hazard ratio of death during dengue infection |  | [1,10] | Uniform(1,10) |  |
| Limit on infection given previous dengue infection |  | (0,1] | Uniform(0,1) |  |
| Initial number of people infected with chikungunya, asymptomatic |  | (0,5000] | Uniform(0,5000) |  |
| Initial number of people infected with chikungunya, symptomatic |  | (0,10000] | Uniform(0,5000) |  |
| Initial number of mosquitos infected with chikungunya |  | (0,20000] | Uniform(0,20000) |  |
| Initial number of people infected with dengue, asymptomatic |  | (0,5000] | Uniform(0,5000) |  |
| Initial number of people infected with chikungunya, symptomatic |  | (0,5000] | Uniform(0,5000) |  |
| Initial number of mosquitos infected with dengue |  | (0,20000] | Uniform(0,20000) |  |

**Table B. Percent of Parameter Sets that Prefer Each Intervention, by Vaccine Cost (C_V_) and Diagnostic Test Cost (C_D_)**

|  | **C_V_=$75.5**  **C_D_ = $8^*^** | **C_V_ = $10**  **C_D_ = $1** | **C_V_ = $9**  **C_D_ = $1** | **C_V_ = $8**  **C_D_ = $1** | **C_V_ = $7**  **C_D_ = $1** | **C_V_ = $6**  **C_D_ = $1** | **C_V_ = $5**  **C_D_ = $1** | **C_V_ = $4**  **C_D_ = $1** | **C_V_ = $3**  **C_D_ = $1** | **C_V_ = $0**  **C_D_ = $0** |
| --- | --- | --- | --- | --- | --- | --- | --- | --- | --- | --- |
| Status Quo | 13 | 10 | 10 | 10 | 9.33 | 8 | 7.33 | 5.33 | 2 | 0 |
| Insecticide | 85.67 | 72.67 | 64 | 54 | 50 | 43 | 35.33 | 22.33 | 9.33 | 0 |
| LLIN | 0 | 0 | 0 | 0 | 0 | 0 | 0 | 0 | 0 | 0 |
| Routine Dengue Vaccination | 0 | 0 | 0 | 0 | 0 | 0 | 0 | 0 | 0 | 0 |
| Routine Dengue Vaccination + Catchup at Ages 10-19 | 0 | 0 | 0 | 0 | 0 | 0 | 0 | 0 | 0 | 0 |
| Routine Dengue Vaccination + Catchup at Ages 10-29 | 0 | 7.33 | 13.33 | 17.67 | 19.33 | 23.67 | 26.33 | 29 | 35.67 | 39.33 |
| Insecticide + Routine Dengue Vaccination | 0 | 0 | 0 | 0 | 0 | 0 | 0 | 0 | 0 | 0 |
| Insecticide + Routine Dengue Vaccination + Catchup for Ages 10-19 | 0 | 8.33 | 9.67 | 13 | 11 | 6.67 | 4.33 | 1.67 | 0.67 | 0 |
| Insecticide + Routine Dengue Vaccination + Catchup for Ages 10-29 | 0 | 0.33 | 1.67 | 4 | 9 | 17.33 | 25.33 | 40.33 | 51.67 | 60.33 |
| LLIN + Routine Dengue Vaccination | 0 | 0 | 0 | 0 | 0 | 0 | 0 | 0 | 0 | 0 |
| LLIN + Routine Dengue Vaccination + Catchup for Ages 10-19 | 0 | 0 | 0 | 0 | 0 | 0 | 0 | 0 | 0 | 0 |
| LLIN + Routine Dengue Vaccination + Catchup for Ages 10-29 | 0 | 0 | 0 | 0 | 0 | 0 | 0 | 0 | 0 | 0 |
| Insecticide + LLIN | 1.33 | 1.33 | 1.33 | 1.33 | 1.33 | 1.33 | 1.33 | 1.33 | 0.67 | 0 |
| Insecticide + LLIN + Routine Dengue Vaccination | 0 | 0 | 0 | 0 | 0 | 0 | 0 | 0 | 0 | 0 |
| Insecticide + LLIN + Routine Dengue Vaccination + Catchup for Ages 10-19 | 0 | 0 | 0 | 0 | 0 | 0 | 0 | 0 | 0 | 0 |
| Insecticide + LLIN + Routine Dengue Vaccination + Catchup for Ages 10-29 | 0 | 0 | 0 | 0 | 0 | 0 | 0 | 0 | 0 | 0.33 |

Preferred strategy is defined as the intervention with the minimum incremental cost/DALY averted less than $18,132. If each incremental cost/DALY averted is greater than the WTP, the status quo is preferred; LLIN = long-lasting insecticide-treated nets; Routine Dengue Vaccination corresponds to vaccination of all 9-year-olds

* Base case

**Table C. One-way Sensitivity Analysis of Insecticide and LLIN Efficacy, Cost, and Coverage**

| **Insecticide** | | | | | | **LLIN** | | | | | |
| --- | --- | --- | --- | --- | --- | --- | --- | --- | --- | --- | --- |
| **Insecticide Cost**  **($/household)** | **Cost per DALY Averted** | **Insecticide Efficacy (%)** | **Cost per DALY Averted** | **Insecticide Coverage (%)** | **Cost per DALY Averted** | **LLIN Cost**  **($/household)** | **Cost per DALY Averted** | **LLIN Efficacy (%)** | **Cost per DALY Averted** | **LLIN Coverage (%)** | **Cost per DALY Averted** |
| 3 | 3,279 | 64 | 3,279 | 1 | 2,244 | 48 | 59,647 | 100 | 43,244 | 1 | 52,644 |
| 4 | 6,180 | 60 | 3,767 | 2 | 2,478 | 46 | 56,918 | 90 | 47,699 | 2 | 54,432 |
| 5 | 9,081 | 50 | 5,307 | 3 | 2,730 | 40 | 48,730 | 85 | 50,321 | 3 | 56,161 |
| 6 | 11,982 | 40 | 7,601 | 4 | 2,996 | 30 | 35,084 | 80 | 53,270 | 4 | 57,874 |
| 7 | 14,883 | 30 | 11,477 | 5 | 3,279 | 20 | 21,438 | 75 | 56,614 | 5 | 59,647 |
| 8 | 17,784 | 25 | 14,512 | 6 | 3,554 | 19 | 20,073 | 71 | 59,647 | 6 | 61,426 |
| 8.5 | 19,234 | 24 | 15,280 | 7 | 3,812 | 18 | 18,708 | 65 | 64,862 | 7 | 63,255 |
| 9 | 20,685 | 23 | 16,102 | 8 | 4,084 | 17 | 17,344 | 60 | 70,029 | 8 | 65,105 |
| 10 | 23,586 | 22 | 17,002 | 9 | 4,366 | 16 | 15,979 | 55 | 76,139 | 9 | 66,996 |
| 11 |  | 21 | 17,996 | 10 | 4,575 | 15 | 14,614 | 50 | 83,472 | 10 | 68,911 |
| 12 |  | 20 | 19,072 | 11 | 4,851 |  |  |  |  | 11 | 70,847 |
| 13 |  | 15 | 26,704 | 12 | 5,128 |  |  |  |  |  |  |
| 14 |  | 10 | 41,799 | 13 | 5,403 |  |  |  |  |  |  |
| 15 |  |  |  | 14 | 5,680 |  |  |  |  |  |  |

LLIN = long-lasting insecticide-treated nets

**Table D. Scenario Analysis of Hypothetical Chikungunya Vaccine**

| **Intervention** | **Total Cost (2015 USD, millions)** | **Incremental Costs (2015 USD, millions)** | **Total DALYs** | **DALYs Averted** | **NMB** | **ICER** |
| --- | --- | --- | --- | --- | --- | --- |
| Routine Chikungunya Vaccination | 751,795  (751,750, 751,837) | 168  (120, 200) | 77,103,002  (77,093,971, 77,111,517) | 181  (120, 278) | -165  (-196, -118) | 928,426 |
| Routine Chikungunya Vaccination + Catchup at Ages 10-19 | 752,436  (752,409, 752,490) | 809  (780, 854) | 77,100,250  (77,091,626, 77,109,883) | 2933  (863, 5444) | -756  (-838, -699) | 275,802 |
| Routine Chikungunya Vaccination + Catchup at Ages 10-29 | 753,037  (753,004, 753,103) | 1410  (1375, 1468) | 77,097,502  (77,087,841, 77,108,514) | 5680  (1476, 10,113) | -1307  (-1427, -1193) | 248,203 |

NMB = Net monetary benefit, calculated assuming a willingness to pay of $18,132 per DALY; ICER = incremental cost per DALY averted, compared to status quo**;** Routine Dengue Vaccination corresponds to vaccination of all 9-year-olds

**Table E. Scenario Analysis of Test and Vaccinate Strategy using the DENV Detect IgG ELISA Dengue Diagnostic Test and Dengvaxia**

| **Intervention** | **Total Cost (2015 USD, millions)** | **Incremental Costs (2015 USD, millions)** | **Total DALYs** | **DALYs Averted** | **NMB** | **ICER** |
| --- | --- | --- | --- | --- | --- | --- |
| Dengue Vaccine- Routine at Age 9 | 751,812  (751,794, 751,918) | 185  (168, 272) | 77,102,960  (77,094,129, 77,111,708) | 236  (14, 1072) | -180  (-267, -149) | 781,786 |
| Dengue Vaccine- Routine at Age 9 + Catch-up at Ages 10-19 | 752,174  (752,101, 752,286) | 547  (468, 650) | 77,100,722  (77,091,627, 77,110,300) | 2475  (194, 8400) | -502  (-629, -353) | 221,040 |
| Dengue Vaccine- Routine at Age 9 + Catch-up at Ages 10-29 | 752,517  (752,314, 752,644) | 890  (681, 1009) | 77,099,299  (77,089,240, 77,109,436) | 3897  (362, 10,069) | -819  (-971, -629) | 228,243 |
| Insecticide + Routine Dengue Vaccine | 751,827  (751,757, 751,956) | 199  (131, 310) | 77,098,529  (77,086,062, 77,109,932) | 4667  (583, 14,400) | -115  (-299, 129) | 42,676 |
| Insecticide + Routine Dengue Vaccine and Catch-up for Ages 10-19 | 752,192  (752,119, 752,323) | 565  (492, 687) | 77,097,004  (77,084,556, 77,109,060) | 6193  (1059, 16,577) | -453  (-659, -191) | 91,250 |
| Insecticide + Routine Dengue Vaccine and Catch-up for Ages 10-29 | 752,537  (752,360, 752,682) | 909  (727, 1046) | 77,095,953  (77,083,486, 77,107,898) | 7243  (1453, 17,512) | -778  (-1002, -519) | 125,550 |
| LLIN + Routine Dengue Vaccine | 751,955  (751,885, 752,076) | 328  (259, 430) | 77,100,616  (77,087,407, 77,110,958) | 2581  (15, 11,711) | -281  (-424, -46) | 127,034 |
| LLIN + Routine Dengue Vaccine and Catch-up for Ages 10-19 | 752,320  (752,243, 752,443) | 692  (615, 808) | 77,098,747  (77,085,793, 77,109,687) | 4450  (226, 15,184) | -612  (-786, -326) | 155,560 |
| LLIN + Routine Dengue Vaccine and Catch-up for Ages 10-29 | 752,663  (752,474, 752,801) | 1036  (841, 1167) | 77,097,481  (77,084,195, 77,109,159) | 5716  (421, 16,586) | -932  (-1127, -651) | 181,183 |
| Insecticide + LLIN + Routine Dengue Vaccine | 751,974  (751,875, 752,113) | 346  (249, 468) | 77,096,976  (77,080,827, 77,109,830) | 6221  (650, 20,447) | -234  (-456, 117) | 55,688 |
| Insecticide + LLIN + Routine Dengue Vaccine and Catch-up for Ages 10-19 | 752,341  (752,242, 752,480) | 713  (616, 845) | 77,095,594  (77,079,713, 77,108,641) | 7602  (1237, 21,251) | -575  (-816, -240) | 93,808 |
| Insecticide + LLIN + Routine Dengue Vaccine and Catch-up for Ages 10-29 | 752,685  (752,520, 752,839) | 1058  (887, 1204) | 77,094,610  (77,079,201, 77,107,514) | 8587  (1519, 22,308) | -902  (-1156, -541) | 123,224 |

LLIN = long-lasting insecticide-treated nets; Routine Dengue Vaccination corresponds to vaccination of all 9-year-olds; NMB = Net monetary benefit, calculated assuming a willingness to pay of $18,132 per DALY; ICER = incremental cost per DALY averted, compared to status quo

**Table F. Scenario Analysis of Test and Vaccinate Strategy with TAK-003 Dengue Vaccine**

| **Intervention** | **Total Cost (2015 USD, millions)** | **Incremental Costs**  **(2015 USD, millions)** | **Total DALYs** | **DALYs Averted** | **NMB** | **ICER** |
| --- | --- | --- | --- | --- | --- | --- |
| Routine Dengue Vaccination | 751,780  (751,767, 751,846) | 152  (139, 203) | 77,102,872  (77,093,953, 77,111,652) | 324  (66, 1248) | -146  (-197, -119) | 469,551 |
| Routine Dengue Vaccination + Catchup at Ages 10-19 | 752,082  (752,050, 752,152) | 454  (422, 516) | 77,099,651  (77,090,456, 77,109,731) | 3545  (854, 9133) | -390  (-486, -256) | 128,107 |
| Routine Dengue Vaccination + Catchup at Ages 10-29 | 752,369  (752,290, 752,449) | 742  (657, 813) | 77,097,636  (77,088,259, 77,108,170) | 5560  (1563, 10,698) | -641  (-766, -520) | 133,367 |
| Insecticide + Routine Dengue Vaccination | 751,795  (751,730, 751,883) | 168  (104, 240) | 77,098,477  (77,085,984, 77,109,902) | 4719  (614, 14,444) | -82  (-229, 157) | 35,494 |
| Insecticide + Routine Dengue Vaccination + Catchup for Ages 10-19 | 752,101  (752,031, 752,189) | 473  (407, 553) | 77,096,221  (77,083,972, 77,108,461) | 6976  (1485, 17,027) | -347  (-516, -94) | 67,845 |
| Insecticide + Routine Dengue Vaccination + Catchup for Ages 10-29 | 752,390  (752,320, 752,487) | 763  (693, 850) | 77,094,680  (77,082,429, 77,107,249) | 8517  (2319, 18,260) | -608  (-789, -370) | 89,548 |
| LLIN + Routine Dengue Vaccination | 751,923  (751,857, 752,003) | 296  (231, 360) | 77,100,551  (77,087,378, 77,110,931) | 2646  (71, 11,772) | -248  (-354, -17) | 111,878 |
| LLIN + Routine Dengue Vaccination + Catchup for Ages 10-19 | 752,227  (752,156, 752,309) | 600  (529, 673) | 77,097,827  (77,084,574, 77,109,102) | 5370  (937, 16,060) | -503  (-643, -233) | 111,711 |
| LLIN + Routine Dengue Vaccination + Catchup for Ages 10-29 | 752,515  (752,440, 752,606) | 888  (813, 970) | 77,096,007  (77,082,984, 77,107,772) | 7190  (1639, 17,670) | -758  (-923, -501) | 123,520 |
| Insecticide + LLIN + Routine Dengue Vaccination | 751,942  (751,848, 752,041) | 315  (223, 398) | 77,096,931  (77,080,815, 77,109,799) | 6265  (679, 20,468) | -201  (-386, 143) | 50,261 |
| Insecticide + LLIN + Routine Dengue Vaccination + Catchup for Ages 10-19 | 752,249  (752,155, 752,346) | 622  (529, 710) | 77,094,870  (77,079,284, 77,108,296) | 8326  (1659, 21,775) | -471  (-674, -141) | 74,653 |
| Insecticide + LLIN + Routine Dengue Vaccination + Catchup for Ages 10-29 | 752,539  (752,449, 752,644) | 911  (824, 1007) | 77,093,414  (77,078,248, 77,106,884) | 9783  (2475, 23,091) | -734  (-945, -415) | 93,171 |

LLIN = long-lasting insecticide-treated nets; Routine Dengue Vaccination corresponds to vaccination of all 9-year-olds; NMB = Net monetary benefit, calculated assuming a willingness to pay of $18,132 per DALY; ICER = incremental cost per DALY averted, compared to status quo

**Table G. Scenario Analysis of More Effective, Less Costly Vaccine and Less Effective, More Costly Insecticide and LLIN***

| **Intervention** | **Total Cost**  **(2015 USD, millions)** | **Incremental Costs (2015 USD, millions)** | **Total DALYs** | **DALYs Averted** | **NMB** | **ICER** |
| --- | --- | --- | --- | --- | --- | --- |
| Status Quo | 751,627  (751,620, 751,645) | **--** | 77,103,196  (77,094,183, 77,111,793) | **--** | **--** | **--** |
| Insecticide | 751,732  (751,696, 751,762) | 105  (70, 117) | 77,100,713  (77,089,369, 77,110,591) | 2484  (178, 8147) | -60  (-114, 77) | 42,245 |
| LLIN | 752,092  (752,057, 752,117) | 465  (430, 473) | 77,101,907  (77,091,158, 77,111,429) | 1289  (0, 6708) | -442  (-472, -308) | 360,598 |
| Routine Dengue Vaccination | 751,697  (751,688, 751,737) | 70  (60, 95) | 77,102,862  (77,093,952, 77,111,651) | 334  (66, 1293) | -64  (-90, -39) | 209,627 |
| Routine Dengue Vaccination + Catchup at Ages 10-19 | 751,830  (751,801, 751,880) | 202  (173, 237) | 77,099,581  (77,090,449, 77,109,646) | 3616  (854, 9250) | -137  (-215, -10) | 55,992 |
| Routine Dengue Vaccination + Catchup at Ages 10-29 | 751,958  (751,923, 752,014) | 331  (290, 372) | 77,097,552  (77,088,206, 77,108,024) | 5644  (1577, 10,761) | -228  (-336, -111) | 58,607 |
| Insecticide + Routine Dengue Vaccination | 751,803  (751,764, 751,854) | 175  (137, 213) | 77,100,460  (77,089,221, 77,110,501) | 2736  (424, 8803) | -126  (-204, 20) | 64,067 |
| Insecticide + Routine Dengue Vaccination + Catchup for Ages 10-19 | 751,937  (751,888, 751,997) | 310  (262, 354) | 77,097,763  (77,086,778, 77,109,126) | 5434  (1298, 13,357) | -211  (-324, -21) | 57,039 |
| Insecticide + Routine Dengue Vaccination + Catchup for Ages 10-29 | 752,067  (752,019, 752,131) | 439  (394, 489) | 77,096,009  (77,085,331, 77,107,557) | 7188  (2026, 14,512) | -309  (-450, -137) | 61,118 |
| LLIN + Routine Dengue Vaccination | 752,163  (752,124, 752,210) | 535  (498, 568) | 77,101,625  (77,090,987, 77,111,166) | 1572  (69, 7027) | -507  (-563, -371) | 340,538 |
| LLIN + Routine Dengue Vaccination + Catchup for Ages 10-19 | 752,296  (752,245, 752,352) | 669  (621, 709) | 77,098,633  (77,087,527, 77,109,423) | 4564  (913, 12,943) | -586  (-687, -387) | 146,552 |
| LLIN + Routine Dengue Vaccination + Catchup for Ages 10-29 | 752,425  (752,377, 752,486) | 797  (750, 845) | 77,096,712  (77,085,666, 77,107,833) | 6484  (1617, 14,224) | -680  (-806, -498) | 122,990 |
| Insecticide + LLIN | 752,198  (752,139, 752,235) | 571  (513, 590) | 77,099,687  (77,087,095, 77,110,564) | 3509  (179, 12,789) | -507  (-586, -283) | 162,702 |
| Insecticide + LLIN + Routine Dengue Vaccination | 752,269  (752,207, 752,327) | 642  (580, 685) | 77,099,463  (77,086,909, 77,110,279) | 3734  (467, 13,212) | -574  (-676, -344) | 171,819 |
| Insecticide + LLIN + Routine Dengue Vaccination + Catchup for Ages 10-19 | 752,404  (752,338, 752,469) | 777  (712, 827) | 77,096,937  (77,084,191, 77,108,763) | 6260  (1332, 16,586) | -663  (-797, -404) | 124,093 |
| Insecticide + LLIN + Routine Dengue Vaccination + Catchup for Ages 10-29 | 752,534  (752,469, 752,603) | 907  (842, 962) | 77,095,250  (77,082,527, 77,107,367) | 7947  (2058, 17,945) | -762  (-921, -518) | 114,073 |

*****Vaccine efficacy = 90%; Vaccine and diagnostic costs at one-third of base case; Insecticide and LLIN costs triple base case; Insecticide and LLIN efficacy half of base case; LLIN = long-lasting insecticide-treated nets; Routine Dengue Vaccination corresponds to vaccination of all 9-year-olds; NMB = Net monetary benefit, calculated assuming a willingness to pay of $18,132 per DALY; ICER = incremental cost per DALY averted, compared to status quo

**Fig A. Calibration results: 300 best-fitting parameter sets**

**
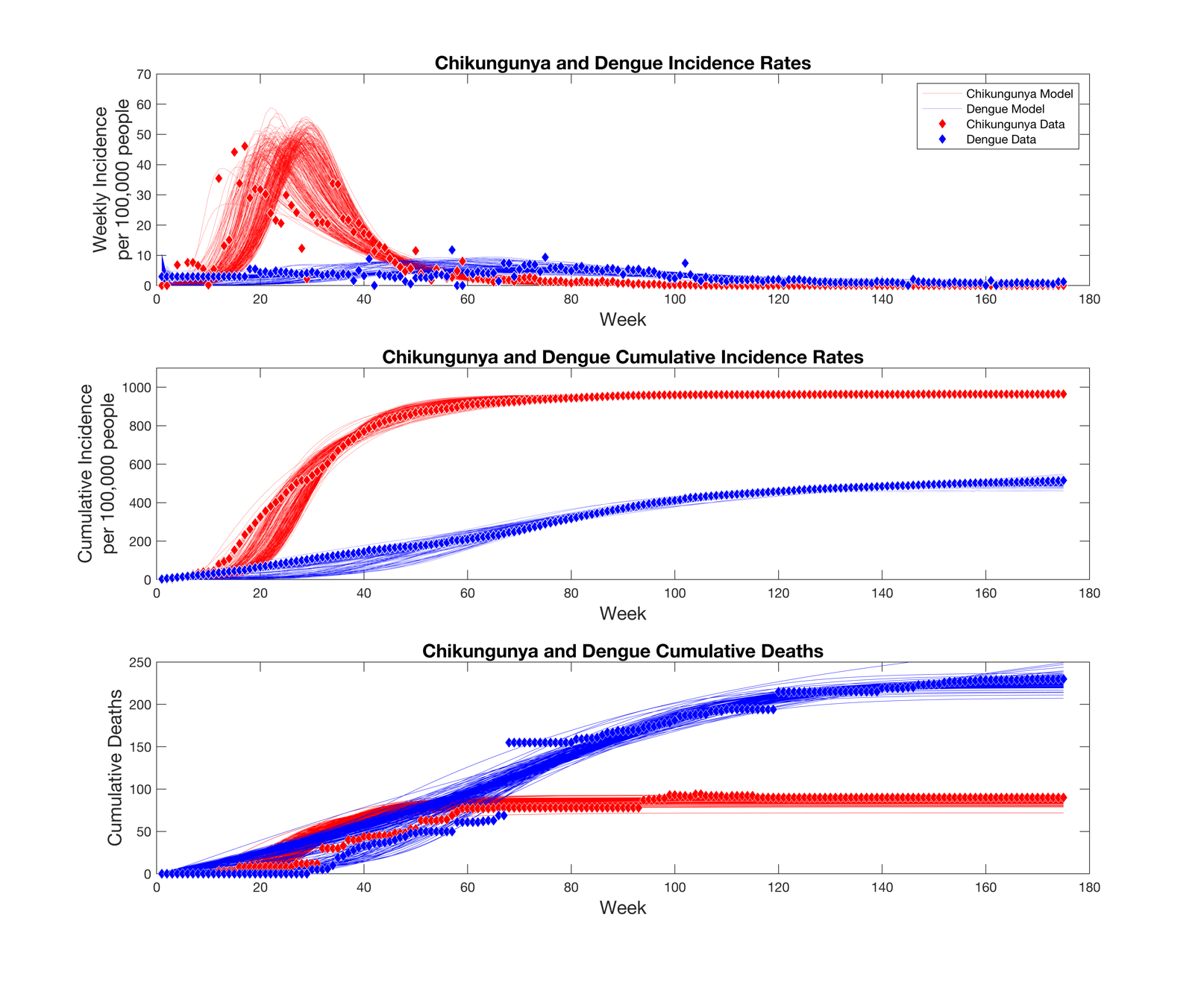
**

**Fig B. Distributions of model parameter values before and after calibration** Blue denotes distribution for each parameter before calibration, orange denotes distribution of each parameter after calibration.

**
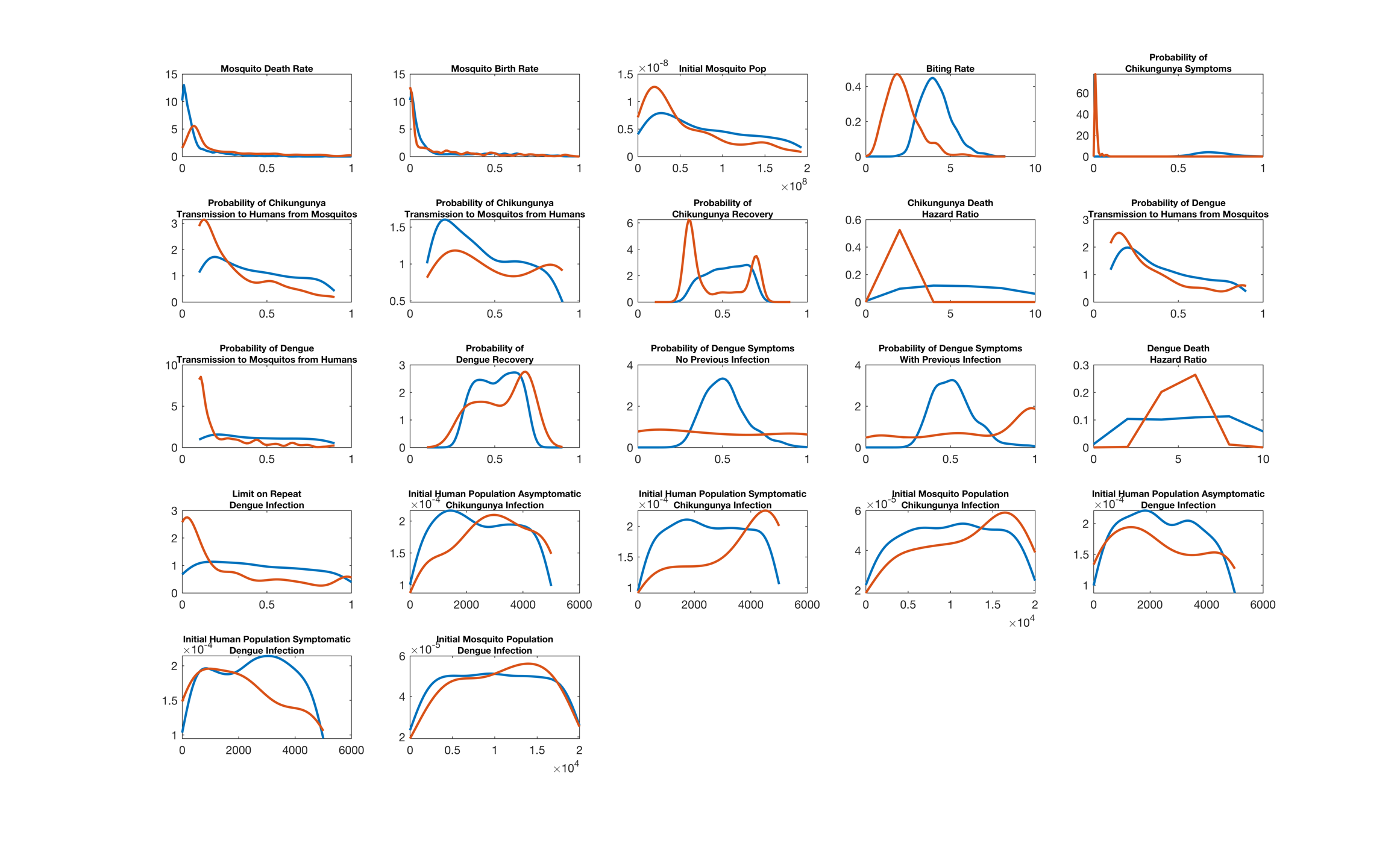
**

**Fig C. Parameter correlation matrix for the 300 best-fitting parameter sets***

*****One-to-one correlations were found for mosquito birth rate/mosquito death rate, dengue death hazard/probability of recovery, and chikungunya death hazard/probability of chikungunya recovery. These come from the necessary steady state needed for each parameter. For example, an increase in the mosquito birth rate also means an increase in mosquito deaths to keep a stable population. An increase in the death hazard during infection is also correlated with an increase in the probability of recovery (which decreases the time an individual is susceptible for higher mortality hazard).

**Fig D. Cumulative incidence of dengue by intervention in Colombia: June 2014 to December 2017** Solid line denotes the mean, and dashed lines denote the range.


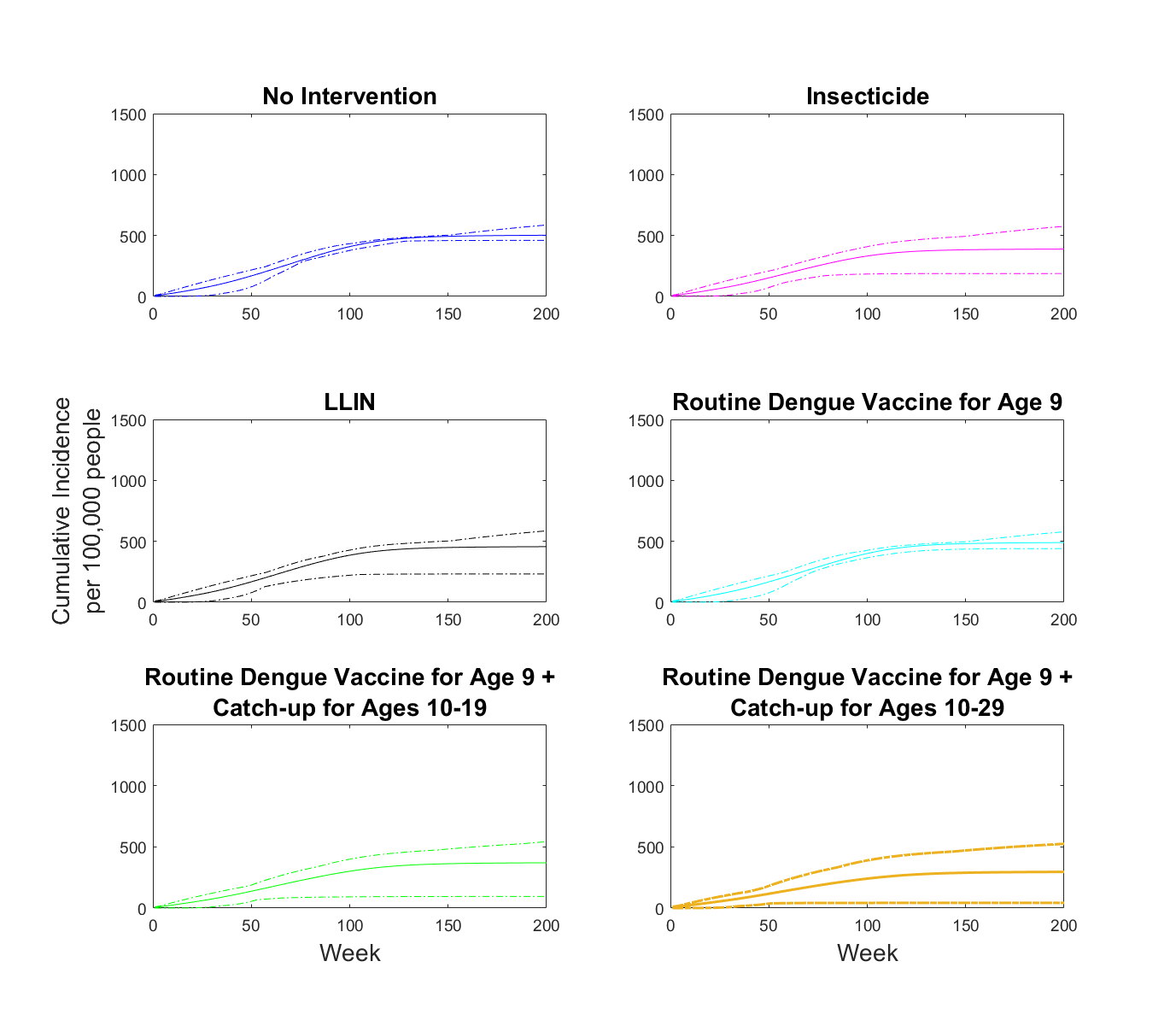


**Fig E. Cumulative incidence of chikungunya by intervention in Colombia: June 2014 to December 2017** Solid line denotes the mean, and dashed lines denote the range.

**
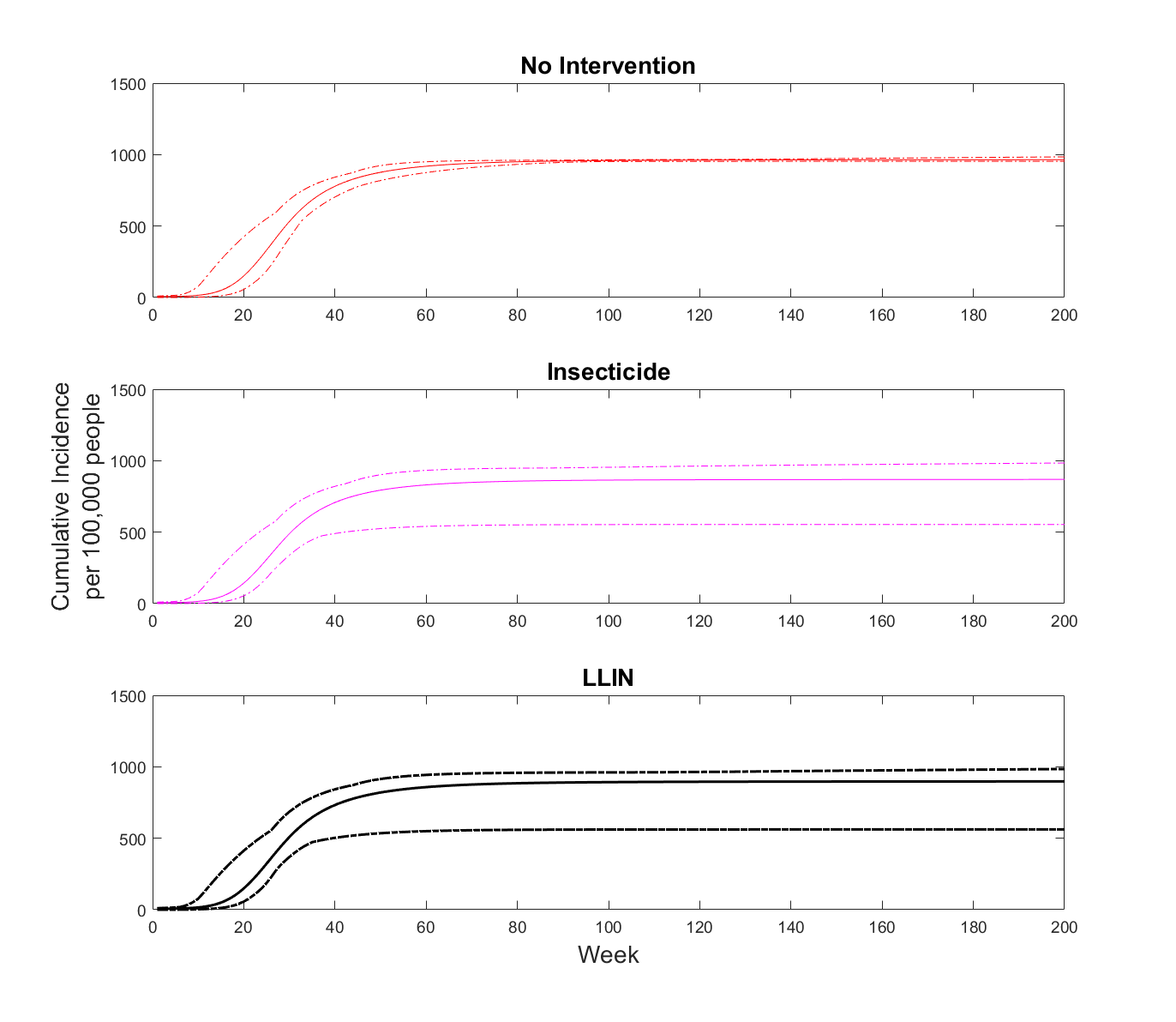
**

**Fig F. Cumulative dengue deaths by intervention in Colombia: June 2014 to December 2017** Solid line denotes the mean, and dashed lines denote the range.

**
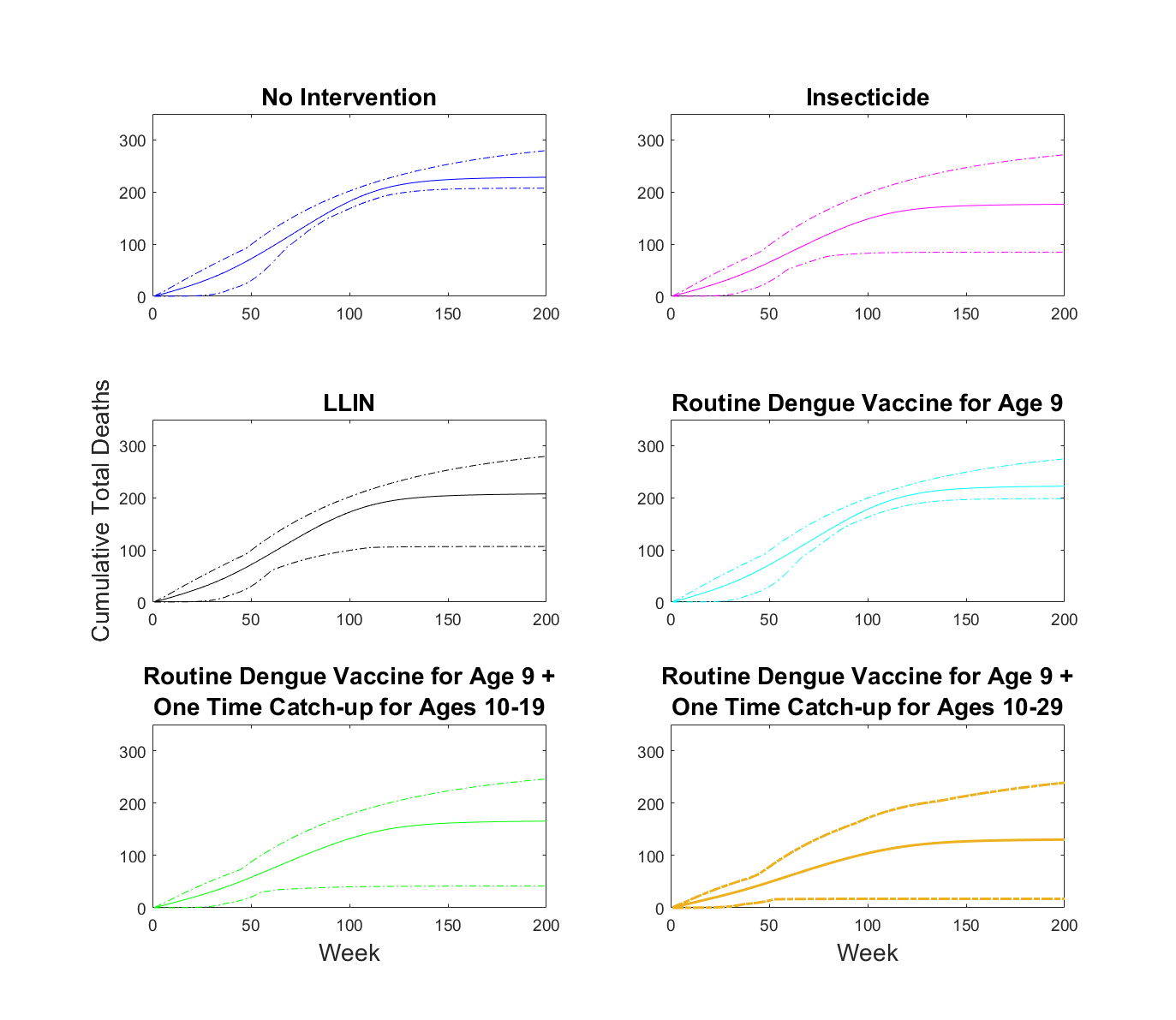
**

**Fig G. Cumulative chikungunya deaths by intervention in Colombia: June 2014 to December 2017** Solid line denotes the mean, and dashed lines denote the range.

**
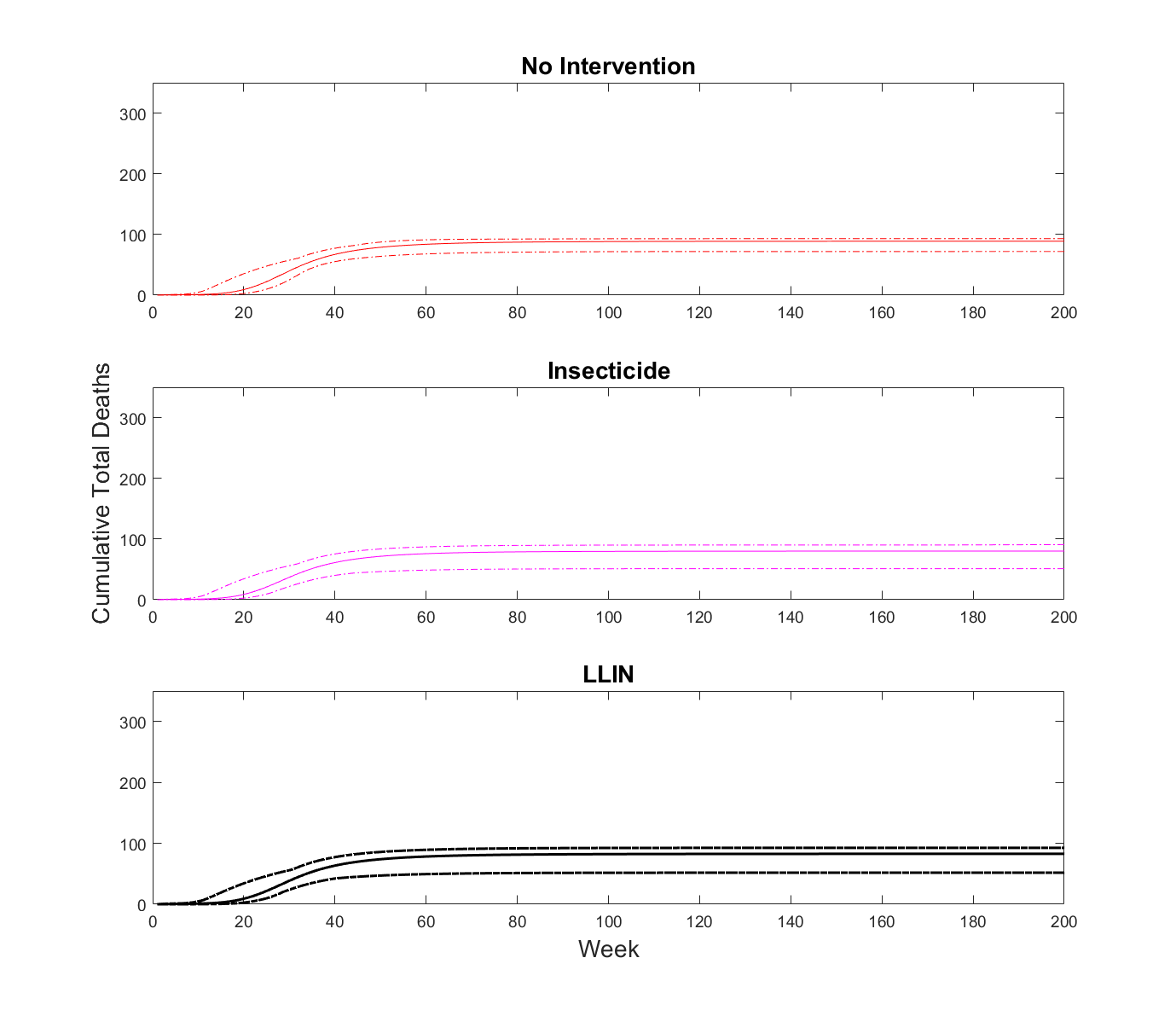
**

References

1. Keeling MJ, Rohani P. Modeling Infectious Diseases in Humans and Animals. Princeton: Princeton University Press; 2008.

2. Claypool AL, Brandeau ML, Goldhaber-Fiebert JD. Quantifying positive health externalities of disease control interventions: modeling chikungunya and dengue. Medical Decision Making (in press). 2019.

3. Briggs AH, Weinstein MC, Fenwick EA, Karnon J, Sculpher MJ, Paltiel AD, et al. Model parameter estimation and uncertainty analysis: a report of the ISPOR-SMDM Modeling Good Research Practices Task Force Working Group-6. Med Decis Making. 2012 Sep-Oct;32(5):722-32.

4. Sculpher MJ, Basu A, Kuntz KM, Meltzer DO. Reflecting uncertainty in cost-effectiveness analysis. In: Neumann PJ, Ganiats TG, Sanders GD, editors. Cost-Effectiveness in Health and Medicine. New York: Oxford University Press; 2016. p. 289–318.

5. Ndeffo-Mbah ML, Durham DP, Skrip LA, Nsoesie EO, Brownstein JS, Fish D, et al. Evaluating the effectiveness of localized control strategies to curtail chikungunya. Sci Rep. 2016 Apr 5;6:23997.

6. Robinson M, Conan A, Duong V, Ly S, Ngan C, Buchy P, et al. A model for a chikungunya outbreak in a rural Cambodian setting: implications for disease control in uninfected areas. PLoS Negl Trop Dis. 2014 Sep;8(9):e3120.

7. Andraud M, Hens N, Marais C, Beutels P. Dynamic epidemiological models for dengue transmission: a systematic review of structural approaches. PLoS One. 2012;7(11):e49085.

8. Clapham HE, Cummings DAT, Johansson MA. Immune status alters the probability of apparent illness due to dengue virus infection: evidence from a pooled analysis across multiple cohort and cluster studies. PLoS Negl Trop Dis. 2017 Sep;11(9):e0005926.
